# Future Climate Predicts Range Shifts and Increased Global Habitat Suitability for 29 *Aedes* Mosquito Species

**DOI:** 10.3390/insects16050476

**Published:** 2025-04-30

**Authors:** Xueyou Zhang, Hongyan Mei, Peixiao Nie, Xiaokang Hu, Jianmeng Feng

**Affiliations:** 1 College of Agriculture and Biological Science, Dali University, Dali 671003, China; 2 Research Center for Agroecology in Erhai Lake Watershed, Dali University, Dali 671003, China; 3 Cangshan Forest Ecosystem Observation and Research Station of Yunnan Province, Dali University, Dali 671003, China

**Keywords:** *Aedes* mosquito species, climate changes, habitat suitability, future scenario, global scale, range dynamics

## Abstract

*Aedes* mosquitoes (Diptera, Culicidae) are the major vectors for many mosquito-borne diseases. Here, we retrieved 878,954 global occurrences of 29 *Aedes* mosquito species and 30 candidate predictors at a global scale. We created a unified frame and built 29 multi-algorithm species distribution models to project the ranges and overlapped them to examine the range-overlap hotspots under future scenarios. We detected expanded ranges in most *Aedes* mosquito species, and a substantial increase in the index of habitat suitability overlap was detected in more than 70% of the global terrestrial area, particularly in Europe, North America, and Africa. We also identified extensive range overlap, which increased in future scenarios. Climatic factors had a more significant influence on range dynamics than other variables. The expanded ranges of most *Aedes* mosquito species and the substantial increase in the overlap index of habitat suitability in most regions suggest globally increasing threats of *Aedes*-borne epidemic transmission. Thus, much stricter strategies must be implemented, particularly in Europe, North America, and Africa. As climate change increases habitat suitability and expands ranges in most *Aedes* mosquito species, mitigating future climate change will be a key approach to combatting their impacts.

## Simple Summary

*Aedes* mosquitoes (Diptera, Culicidae) are key vectors of numerous mosquito-borne diseases. In this study, we analyzed 878,954 global occurrences of 29 *Aedes* species alongside 30 environmental predictors. Using multi-algorithm species distribution models, we projected future ranges and identified overlap hotspots. Most species exhibited expanded ranges, with habitat suitability overlap increasing across 70% of the global land area, particularly in Europe, North America, and Africa. Climate factors played a dominant role in these changes. The rising overlap index and expanding ranges indicated a growing risk of *Aedes*-borne epidemics, necessitating stricter control measures, particularly in high-risk regions. Climate change is a key driver of increased habitat suitability, emphasizing the need for mitigation strategies to limit its impact on mosquito-borne disease transmission.

## 1. Introduction

More than ½ of the global population is at risk of mosquito-borne diseases [1]. *Aedes* mosquitoes (Diptera, Culicidae) are key vectors for yellow fever, dengue, Zika, and chikungunya [2,3,4,5], which persist despite efforts by the World Health Organization and others, with only small-scale control successes [6,7,8]. These mosquitoes also affect domestic and farm animals, causing major economic losses [9,10,11]. Controlling the spread of *Aedes* is key. Larger species ranges indicate greater risks to human, domestic, and farm animal health, with expansions signaling worse future impacts [7,12,13,14]. Overlapping ranges among *Aedes* species require stricter, broader strategies. Mapping distributions, overlaps, and potential expansion could inform effective control [15,16,17,18,19]. Research has emphasized *Ae. albopictus* and *Ae. aegypti*, the leading arboviral vectors, but has often overlooked other *Aedes* species or used inconsistent methods. For example, Echeverry-Cárdenas et al. [20] studied future range shifts of *Ae. albopictus*, while Nie and Feng [21] analyzed those in *Ae. aegypti*, yet differences in scale, predictors, and algorithms hindered risk comparisons. A unified framework assessing future range shifts and overlaps across *Aedes* species would enhance risk evaluation and disease control strategies.

Climate conditions are closely associated with the physiological activities and life history of *Aedes* mosquitoes [22,23,24]. Cai et al. reported that temperature exerts a significant effect on the development and physiological stages of *Ae. albopictus* [25], and Lusekelo et al. observed a close association between temperature and the growth of an *Aedes* mosquito population [26]. Many studies have indicated that climate change could play a substantial role in the range shifts of *Aedes* mosquitoes, and their target species mainly include *Ae. aegypti* [14,16], *Ae. albopictus* [21], *Ae. japonicus* [27,28], and *Ae. vexans* [29,30]. For example, Georgiades et al. reported the strong influence of winter and summer temperatures on the range expansion of *Ae. albopictus* [31], which was consistent with recent findings [17,21]. These studies offer updated insight into the roles of climate change in the range shifts in *Aedes* mosquito species. However, the roles of climate change in the range shifts of most *Aedes* mosquito species and their overlapping ranges have not been investigated.

Anthropogenic disturbances, particularly changes in land use, can shift the habitats, predators, and hosts of *Aedes* mosquitoes, which could result in shifts in the range and range overlap to search for alternative habitats and host sources or to escape from predators [32]. Zahouli et al. observed the strong roles of anthropogenic disturbances in the distribution of *Aedes* mosquitoes in Côte d’Ivoire [33], and Dickens et al. argued that human activities promote shifts in the distribution of *Ae. albopictus* and *Ae. aegypti* [34]. Although anthropogenic disturbances and climate change could trigger changes in the range shifts of *Aedes* mosquitoes, their relative effects on range shifts are controversial. For instance, Liu et al. reported that climate change has a greater effect on the range dynamics of two *Aedes* mosquito species compared to human-driven factors [35], whereas Dickens et al. detected the opposite [34]. Thus, the relative role of human disturbances and climate change in the shifts of the distribution patterns of most *Aedes* mosquitoes and their overlapping ranges require more attention.

Topographical patterns, such as mountains and deep valleys, can change the distribution of energy and water resources, which are closely linked to habitat diversity [36,37]. Additionally, rugged topographical patterns act as barriers against the dispersal of many species [38]. Therefore, although climate change can strongly affect changes in the geographic range of *Aedes* mosquito species, the influence of topographical variables on the changes in the geographic ranges of *Aedes* mosquito species cannot be overlooked. For example, Lippi et al. observed a strong role for elevation in the potential range of *Ae. aegypti* in Ecuador [39]. However, until now, the roles of topographical variables in the distribution shifts of *Aedes* mosquitoes and their overlapping ranges, relative to those of climate change, have been little investigated [40,41].

Here, using climate change, topography, and human disturbance data layers, we created models predicting range shifts and their overlap for 29 *Aedes* species within a unified frame. We assumed that the relative roles of human disturbance, topography, and climate change in the future range and range overlap shifts of *Aedes* mosquitoes are species-specific. We hope our study offers novel insight into preventing or mitigating the future impacts of *Aedes* mosquito species.

## 2. Materials and Methods

### 2.1. Retrieving Records of the Target Species

**Figure 1:**
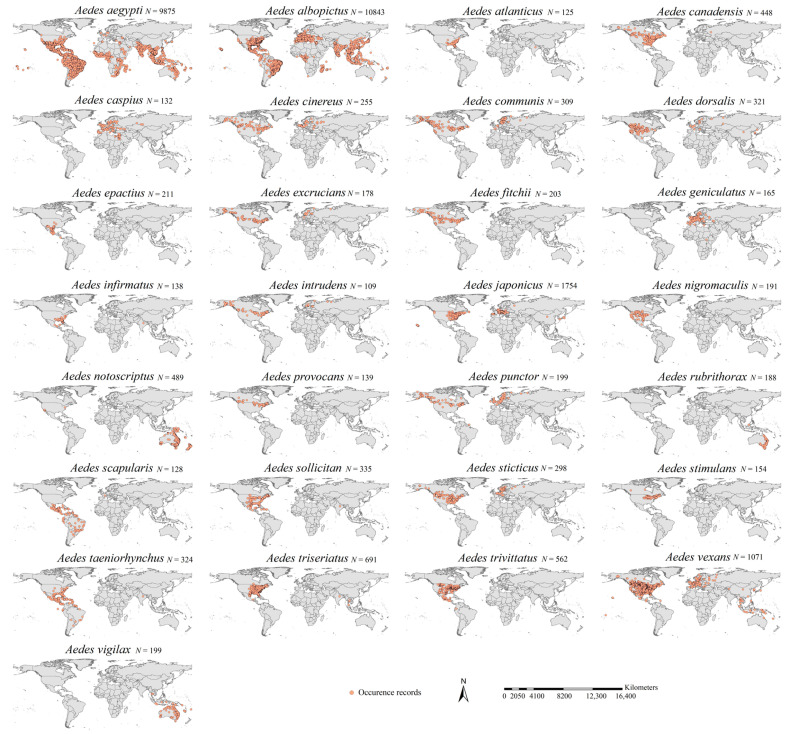
Occurrence maps of the 29 major *Aedes* mosquito species. The occurrences were obtained through extensive surveys of the literature and online datasets. *N* in the figure indicates the number of occurrences of each species after the spatial thinning. In total, we retrieved 30,074 occurrence points after the spatial thinning.

We conducted a survey on the taxonomical system, distributions, and impacts of *Aedes* mosquito species [42,43,44,45,46] and retrieved a list of common *Aedes* mosquito species worldwide. Their occurrence records were retrieved through a survey of the literature [5,29,35,47,48,49,50,51,52,53,54,55,56,57,58] and online data sources (Global Biodiversity Information Facility, www.gbif.org; Centers for Disease Control and Prevention—Aedes Mosquito Surveillance Data, www.cdc.gov; Mosquito Distribution Maps, svs.gsfc.nasa.gov; The European Centre for Disease Prevention and Control—Aedes Mosquito Mapping, www.ecdc.europa.eu. All accessed on 21 October 2023). In total, we obtained 954,717 records of common *Aedes* mosquito species. We preliminarily built an occurrence dataset for each species. As recommended by Zhou et al. [59], we only retained the records for each species with geographical coordinate uncertainty <5 km. Then, we spatially rarefied the records for each species; i.e., only one record in each 10 × 10 km grid cell was retained. To guarantee that the records reliably reflected the niches or adaptations to environmental conditions, we used a threshold of 100 spatially rarefied records to select the *Aedes* species. Finally, we retrieved 29 *Aedes* species, with 878,954 and 30,074 occurrences before and after the spatial rarefication, respectively (Figure 1).

### 2.2. Variables Included in the Models

In total, we compiled 30 variables to calibrate the habitat suitability index (HSI) and the range of each target species, including climate (19), anthropogenic disturbances (8), and topography (3). To determine the climatic variables for the current scenarios, we downloaded monthly temperature and precipitation datasets from 1990 to 2020 at a spatial resolution of 2.5 arc minutes from the Climate Research Division (https://crudata.uea.ac.uk/, accessed on 20 October 2023). Biovarcs [60] was used to determine the values of the 19 climate variables under the current scenarios, including eight temperature factors and eleven precipitation variables, which was consistent with the future 2100 scenarios downloaded from Worldclim [60] (www.worldclim.org, accessed on 20 October 2023). The climate datasets under the future scenarios were derived from the two robust and complementary global circulation models (GCMs), namely, FIO ESM 20 (FIO) and MPI ESM1 2 HR (MPI) [61]. Additionally, we employed two shared socioeconomic pathways (SSPs) for the scenarios, namely, SSP126 and SSP585, which represent optimistic and pessimistic future climate change projections, respectively. Therefore, we had five scenarios and five sets of climatic predictors, i.e., datasets under the current condition (scenario), datasets under the SSP126 scenario determined by GCM FIO ESM 2 0 (F126), datasets under the SSP585 scenario determined by GCM FIO ESM2 0 (F585), datasets under the SSP126 scenario determined by GCM MPI ESM1 2 HR (M126), and datasets under the SSP585 scenario determined by GCM MPI ESM1 2 HR (M585). All predictors of anthropogenic disturbances for the current and future scenarios were represented by eight land-use types, sourced from the Land Use Harmonization dataset (LUH2, available at https://luh.umd.edu/, accessed on 25 October 2023). They had an initial spatial resolution of 1/4 arc degrees and were resampled into a spatial resolution of 2.5 arc minutes. The LUH2 generated two sets of future land-use data, including projections for the SSP126 and SSP585 scenarios in the year 2100. We downloaded a digital elevation model from Worldclim [61] at a 30 s spatial resolution, from which we calculated the topographical factors of slope, elevation, and aspect. The data were later resampled to a 2.5 arc-minute spatial resolution. All topographical predictors remained constant under all scenarios.

### 2.3. Reducing Collinearity

We developed initial species distribution models (SDMs) to calculate the importance scores (ISs) of each predictor for each species (Table S1). In our models, the importance of the predictor was calculated by shuffling each variable, comparing predictions, and using 1 minus the correlation as the score. When data obeyed a normal distribution, Pearson’s correlation coefficient of |0.7| was used as the threshold for identifying strong collinearity between the predictor pairs [62], or Spearman’s correlation coefficient was adopted (Table S2). Variables with a lower IS were removed if significant collinearity was detected between predictor pairs. The reserved variables were inputted into the baseline SDMs.

### 2.4. Predicting Habitat Suitability and the Ranges

We developed and trained 29 baseline SDMs at a global scale to project the HSI and the individual ranges of the 29 *Aedes* mosquito species. We separately predicted the habitat suitability maps and range maps of each *Aedes* mosquito species using the R package Biomod2 V.4.1.2, an ensembled SDM platform [63]. As suggested by Barbet-Massin et al. [64], pseudo absences were generated using a three-round random selection process (PAs): 1000 PAs were randomly generated when the number of *Aedes* species records was <1000, or the number of PAs generated equaled that of the *Aedes* mosquito species occurrences. Our models exported five maps of the HSI for each *Aedes* mosquito species separately under the five scenarios. We adopted the threshold of maximum sensitivity–specificity sum [65] to separately calculate the range of each *Aedes* mosquito species under the five scenarios. Regions with an HSI higher than this threshold were considered potential ranges. The validation of five cross repetitions was used to evaluate the robustness of the SDMs; i.e., 70% of the records were randomly selected to develop the SDMs, while the remaining records were used to evaluate the reliability of the model [66]. As suggested by Nie and Feng [21], models with a true skill statistic (TSS) > 0.6 or an area under the curve (AUC) > 0.8 were included in the ensemble SDMs (Table S3).

### 2.5. Investigating the Habitat Suitability Dynamics

The dynamics of the habitat suitability index (HSI) of each *Aedes* mosquito species were calibrated by subtracting the HSI maps under the future scenarios from those under the current scenario. We also developed and calculated the overlap index of habitat suitability (OIHS) for each scenario by separately overlapping the HSI maps of the 29 *Aedes* mosquito species. Next, we created maps showing changes in the OIHS for the 29 *Aedes* mosquito species by calculating the difference between the current OIHS maps and the future projections.

### 2.6. Investigating Range Shifts and Range Overlaps

We examined the range dynamics of each of the 29 *Aedes* mosquito species individually based on their ranges under the future and current scenarios. Their range shifts were measured by the range similarity index (*RSI*) and the range ratio index (*RRI*). The *RRI* was created to calibrate shifts in range size:

$$RRI=\frac{RFS}{RCS},$$

where *RFS* and *RCS* are the ranges under the future and current scenarios, respectively.

*RSI* was developed to calculate the changes in the range positions:

$$SI=\frac{2SR}{RCS+RFS},$$

where *SR* is the range shared by *RFS* and *RCS*. If *RSI* is >0.5, *RCS* and *RFS* share similar range positions.

Additionally, we separately overlapped the potential ranges of the 29 species in each scenario. The pixels of the potential range of a species were assigned a value of one under each scenario to represent the potential range of a species. For example, if a pixel was overlapped by the potential ranges of eight species, this pixel was assigned eight. Then, to explore the shifts in the range overlaps, the four maps of the overlapped ranges under future scenarios were subtracted from the current ones, separately.

Of note, the unified framework in our study meant the same spatial scale, data sources, candidate predictors, models, and indices for range dynamics, which, to a certain extent, could make our projection comparable among all of our target species.

## 3. Results

### 3.1. Model Performance

Our models performed well. The AUC values for the 29 baseline SDMs of the *Aedes* mosquito species were 0.90–0.97, with a mean value of 0.95 ± 0.02 (Table S4). Similarly, the true skill statistic (TSS) values were 0.85–0.97, averaging 0.93 ± 0.04 (Table S4). These consistently high AUC and TSS values confirm the strong reliability and accuracy of our SDMs.

### 3.2. Major Variables Included in the Models

**Figure 2:**
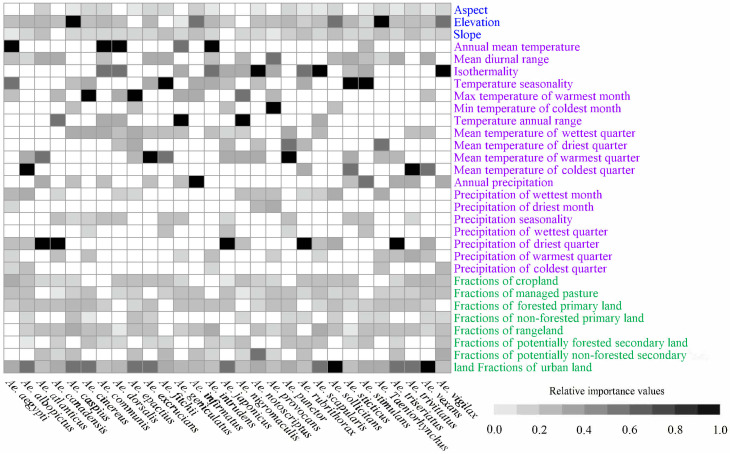
The relative importance of the factors in our baseline models. Topographical, land use, and climatic factors are presented in blue, green, and purple, respectively. The blank space signifies that the variables were not incorporated into the baseline models. Additionally, for each species individually, we utilized the maximum–minimum method to standardize all importance scores. For most major *Aedes* mosquito species, climatic predictors showed higher importance scores than others.

The major factors responsible for the habitat suitability and ranges varied with species (Table S5 and Figure 2). For example, the top three predictors responsible for the ranges of *Ae. aegypti* were the mean annual temperature (IS = 0.253), temperature seasonality (0.114), and the fraction of urban land (0.072), while those for *Ae. fitchii* were temperature seasonality (0.389), mean temperature of the warmest quarter (0.333), and mean diurnal range (0.134) (Figure 2 and Table S5). Additionally, the climatic, topographical, and human disturbance predictors at the category level had the highest ISs in 25, two, and two *Aedes* mosquito species, respectively, and they also appeared 64, 5, and 18 times in the list of the top three predictors responsible for their potential ranges (Figure 2). In summary, climatic predictors played much more significant roles in inducing the range shifts of the 29 *Aedes* mosquito species compared to human disturbances and topography.

### 3.3. Habitat Suitability and the Shift Patterns

The spatial distributions of the HSI varied depending on the scenario. A high HSI of *Ae. cinereus* under the F126 scenario was detected in Western Europe and Western and Eastern Canada, while the HSIs in these regions were substantially lower under the F585 scenario than under the F126 scenario (Figure S1). The HSI spatial patterns were species-specific. For example, the high HSI of *Ae. infirmatus* under the F585 scenario was primarily observed in the USA, while the high HSI of *Ae. caspius* under the F585 scenario was largely projected in Europe (Figure S1).

**Figure 3:**
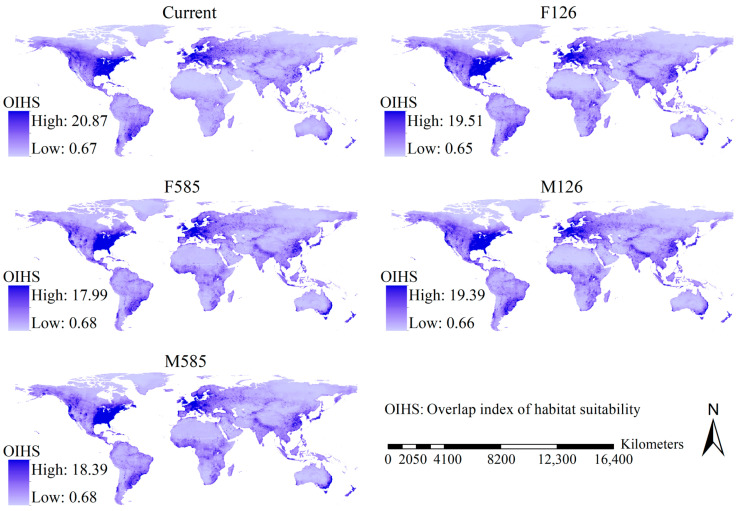
Maps of overlap index of habitat suitability. A significant overlap in the habitat suitability index across the five climate change scenarios was primarily observed in North America and Europe.

Although the geographical patterns of the OIHSs of the 29 *Aedes* mosquito species varied slightly with the scenario, the main body of the high OIHS regions was detected in North America and Europe, and high OIHSs were also detected in East China, Japan, the Korean peninsula, southeastern South America, southeastern Australia, and New Zealand (Figure 3).

**Figure 4:**
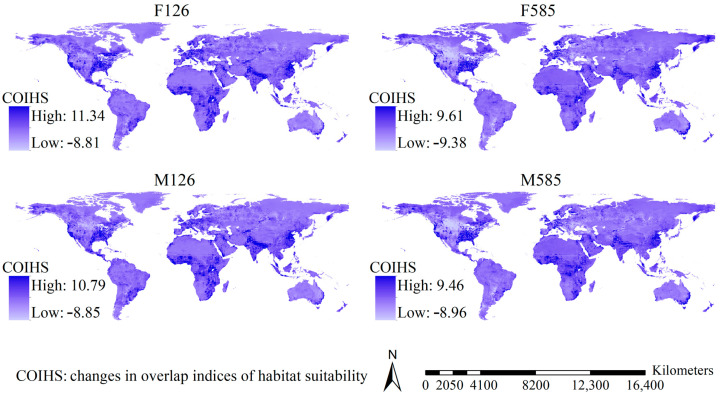
Shifts in the index of habitat suitability overlap of the 29 major *Aedes* mosquitoes under the future scenarios. The substantial increase in the index of habitat suitability overlap under future scenarios was largely predicted in North America and Europe.

Although the shifts in the OIHSs varied slightly with the scenario, major regions showing substantial increases in the OIHSs in the future were largely detected in eastern and western regions of North America and Western Europe, and substantial increases in the OIHSs were also projected in Africa except the desert regions, East China, and tropical regions in Asia as well as the southeastern coastline of Australia, while the regions showing substantial decreases in the OIHSs were mainly scattered in the mid-high latitudinal regions of North America, East Europe, Australia, and Russia (Figure 4). Moreover, areas with increases in the OIHSs covered 104.69, 96.29, 103.43, and 97.44 million km^2^ under the F126, F585, M126, and M585 scenarios. In other words, 77.55%, 71.32%, 76.61%, and 72.18% of the global terrestrial area (except Antarctica) could show higher OIHSs for the 29 *Aedes* mosquito species in the future.

### 3.4. Potential Ranges, Range Overlaps, and Their Shifts

The thresholds of the maximum sensitivity–specificity sum varied between 0.24 and 0.84 (Table S6) and changed with the species and scenario (Table S6). For instance, the thresholds of maximum sensitivity–specificity sum were, respectively, 0.74, 0.72, and 0.73 for *Ae. aegypti*, *Ae. cinereus*, and *Ae. stimulans* under the current scenario, and under F126 and M585, the thresholds of the maximum sensitivity–specificity sum for *Ae. punctor* were 0.59 and 0.49 (Table S6).

**Figure 5:**
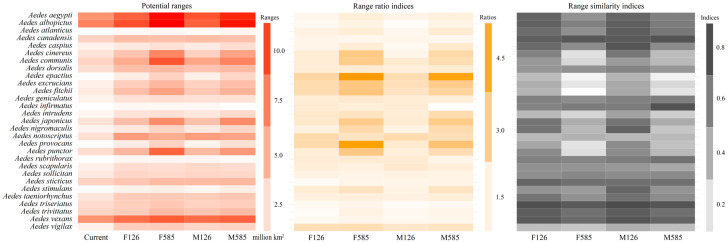
Range shifts in the 29 major *Aedes* mosquitoes in the future. Potential ranges are in red. The range ratio index is in yellow and the range similarity index is in grey. Under most scenarios, the largest ranges under most scenarios are largely observed in *Ae. albopictus*, *Ae. aegypti*, *Ae. trivittatus*, *Ae. communis*, and *Ae. vexans*. Additionally, *Ae. epactius*, *Ae. excrucians*, and *Ae. intrudens* had the largest range ratio indices under most scenarios; *Ae. excrucians* and *Ae. intrudens* had lower range similarity indices than the others.

The ranges in the *Aedes* species were species-specific (Figure S2). For example, the range of *Ae. vexans* under the F126 scenario was largely in North America, Europe, and Africa, and covered 8.25 million km^2^ (Figure 5 and Figure S2), while that of *Ae. infirmatus* was projected in the southeastern USA and covered only 1.03 million km^2^ (Figure 5 and Figure S2). Moreover, the range depended on the scenario. For example, the current range of *Ae. intrudens* was detected in the eastern part of the border between Canada and the USA and covered 1.13 million km^2^, while the ranges under the F126 scenario were mostly in Canada and covered 2.20 million km^2^ (Figure 5 and Figure S2).

The range sizes for the 29 *Aedes* mosquito species varied from 0.56 to 7.45, from 0.89 to 9.55, from 0.97 to 11.24, from 0.79 to 9.41, and from 0.60 to 10.97 million km^2^ under the current, F126, F585, M126, and M585 scenarios, respectively (Figure 5). For most scenarios, the most extensive ranges were observed for *Ae. albopictus*. *Ae. aegypti*, *Ae. punctor*, *Ae. communis*, and *Ae. vexans*. The smallest potential ranges were projected for *Ae. infirmatus*, *Ae. atlanticus*, and *Ae. rubrithorax*, with *Ae. infirmatus* being projected to show the smallest ranges under all scenarios (Figure 5). The paired sample *t*-test indicated that the future ranges of *Aedes* mosquitoes were larger than current ones (*p* < 0.01).

The shifts in the geographic ranges among *Aedes* species varied significantly depending on the species (Figure 5). For example, the range ratio indices (range similarity indices) for *Ae. canadensis* and *Ae. excrucians* were 1.32 (0.78) and 3.23 (0.24), respectively, under the M585 scenario (Figure 5). Additionally, the range shifts in the *Aedes* species were scenario-specific. For example, the range ratio indices (range similarity indices) for *Ae. japonicus* were 1.82 (0.64) and 3.06 (0.42) under the M126 and M585 scenarios, respectively. *Ae. epactius*, *Ae. excrucians*, and *Ae. fitchii* had a frequency of four in the list of the top five range ratio indices in the future, while these were *Ae. vexans* and *Ae. triseriatus* for the lowest range ratio indices (Figure 5). *Ae. canadensis*, *Ae. vexans*, and *Ae. triseriatus* each appeared four times in the rankings of the top five indices of range similarity for future projections, while these were *Ae. fitchii* and *Ae. epactius* for the lowest range similarity indices (Figure 5).

All *Aedes* species were predicted to show larger future ranges (i.e., all range ratio indices > 1). The range ratio indices varied from 1.16 to 2.62, 1.34 to 4.99, 1.17 to 2.46, and 1.02 to 4.64 under the F126, F585, M126, and M585 scenarios, respectively (Figure 5). Range similarity indices were projected to vary from 0.34 to 0.79, 0.09 to 0.76, 0.36 to 0.81, and 0.14 to 0.78 under these scenarios, respectively. Most range similarity indices of the future 29 *Aedes* species were >0.5, i.e., 69.0%, 55.2%, 72,4%, and 51.7% of all target species under the scenarios (Figure 5).

**Figure 6:**
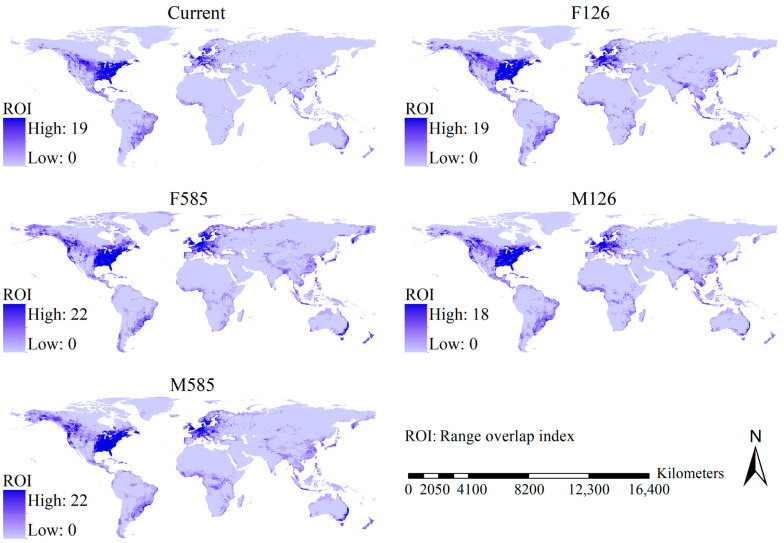
Range overlaps of the 29 major *Aedes* mosquitoes under the five scenarios. A high range overlap was largely detected in North America and Europe. The pixels of the potential range of a species were collectively assigned a value under each scenario to represent the potential range of a species.

**Figure 7:**
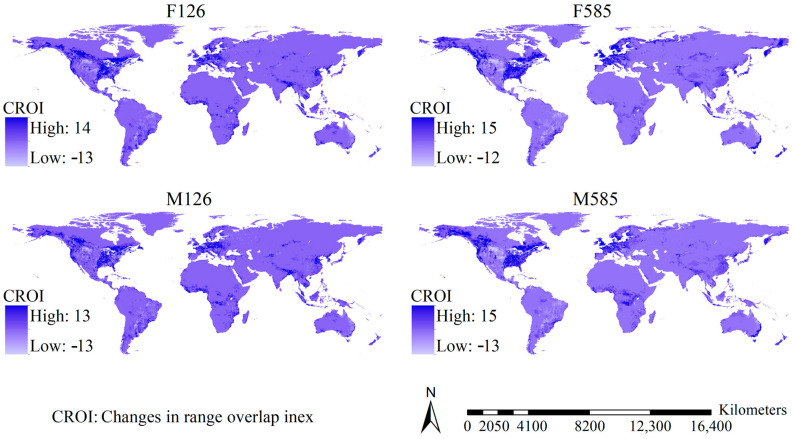
Changes in the range overlaps of the 29 major *Aedes* mosquitoes under current–future scenarios. Substantial increases in the range overlaps were largely detected in the USA (except the desert regions), the southwestern and southeastern regions of Canada, West Europe, the tropical regions of Africa and Asia, East China, Japan, the Korean Peninsula, the southern part of South Africa, as well as the southeastern coastline of Australia.

High range overlaps of the 29 *Aedes* species were observed in Europe and North America and were also projected to scatter in East Asia, northwestern North America, southeastern South America, southeastern Australia, and Tasmania (Figure 6). Roughly consistent with the shifts in OIHSs, under current–future scenarios, we detected substantial increases in the range overlaps in the USA except the desert regions, the southwestern and southeastern regions of Canada, West Europe, the tropical regions of Africa and Asia, East China, Japan, the Korean Peninsula, the southern part of South Africa, as well as the southeastern coastline of Australia, while regions showing substantial decreases in the OIHSs were mainly scattered in the desert regions of North America, Brazil, and the eastern coastline of Australia (Figure 7).

## 4. Discussion

In the present study, we created a unified scheme and developed 29 multi-algorithm SDMs to examine shifts in the global ranges of 29 *Aedes* mosquito species and their overlapping ranges under future climate scenarios. We detected future expansion of the ranges of most of the *Aedes* mosquito species. Moreover, we detected increases in the index of habitat suitability overlap of the 29 *Aedes* species in more than 70% of the global terrestrial area except Antarctica. Consequently, the effects of *Aedes*-borne epidemics on human health and livestock breeding, such as yellow fever, dengue, Zika virus infection, and chikungunya, will increase in the future, so much stricter strategies will be needed, potentially suggesting enormous economic losses in the future. Our observations are supported by several studies on one or more *Aedes* mosquito species [21,67,68,69]. However, different from these studies, we created a unified scheme and developed 29 SDMs to examine the global range shifts in 29 *Aedes* mosquito species individually. This approach helped us to compare the future risk of *Aedes* mosquitoes in terms of the range dynamics to identify high-risk species. Additionally, we also identified the hotspots of their overlapping ranges and spatial shifts under current–future scenarios, which helped us detect the priority regions with the highest threats in the future. Hence, our results offer novel information with which it to devise strategies against the future impacts of *Aedes* mosquitoes.

Climatic and anthropogenic factors play essential roles in the range shifts of *Aedes* mosquito species [34]. Nevertheless, the relative effects on the range dynamics are controversial. For example, Nie and Feng observed more significant effects of climatic variables on the range shifts in *Ae. albopictus* and *Ae. aegypti* [21], whereas Dickens et al. reported the opposite result [34]. The influence of human disturbances versus climatic predictors on range dynamics differs depending on the scale of analysis; i.e., human disturbance variables exhibit stronger effects at a smaller scale, whereas climatic factors show stronger influences at a larger scale [70]. Our global scale study detected stronger roles for climatic predictors in the range shifts of most *Aedes* species (24/29) relative to those of the anthropogenic variables, suggesting that the effects of climatic and human-driven factors on the range dynamics of most *Aedes* mosquito species vary significantly depending on the spatial scale.

Topographical factors are closely associated with the barrier effects against species dispersal [15,36,37]. Nevertheless, we showed that topographical predictors exhibited weaker effects on the range shifts of most of the *Aedes* mosquito species relative to the climatic factors. This may be closely associated with the strong role of human introduction in their proliferation. As many studies have indicated, the transportation of used tires has played a strong role in the proliferation of *Aedes* mosquito species [71,72,73]. For example, Hawley et al. detected that transporting used tires promoted the introduction of *Aedes* species from northern Asia into North America [74]. Accordingly, human introduction, particularly used tire transportation, can overcome topographical barriers through a human-mediated transportation network. Therefore, stricter strategies are necessary for combatting invasion, even in regions characterized by rugged topographical patterns. Virkkala et al. reported a strong role of elevation in the potential range of *Ae. aegypti* in Ecuador in a small spatial scale study [38]. Elevation could be a synthetic proxy for climatic conditions and could play an important role on a smaller scale, whereas it might not necessarily hold on a larger scale [75,76]. Thus, the roles of topographical variables in the range shifts of the *Aedes* mosquito species could be largely overshadowed by human introduction and could also vary with the spatial scale, though further investigations should be conducted to evaluate this hypothesis.

Our study detected a substantial role for climate change in the global range shifts of most target species. Therefore, the climate change-induced range expansion of *Aedes* mosquitoes might potentially promote global *Aedes*-borne epidemics. Many studies on the range dynamics of specific *Aedes* mosquito species support our conclusions [13,67,69]. For example, Nie and Feng identified substantial climate-induced global range expansion in *Ae. albopictus* and *Ae. aegypti* [21]. Compared with these studies, our unified scheme study on 29 *Aedes* mosquito species delineated a general pattern of range expansion under future climate change scenarios. These observations suggest that mitigating climate change is an essential strategy for controlling global *Aedes*-borne epidemics.

We projected high OIHSs under all scenarios in North America, Europe, East China, Japan, the Korean Peninsula, southeastern South America, southeastern Australia, and New Zealand, suggesting that these regions could be more strongly affected by the *Aedes* mosquito species relative to other regions. Additionally, these regions were also the main body of the range overlap hotspots of the 29 *Aedes* mosquito species under all scenarios, suggesting that most *Aedes* mosquito species could occur in these regions. Therefore, these regions could be priority regions for controlling the effects of the *Aedes* mosquito species.

Our research revealed that *Ae. aegypti*, *Ae. Albopictus*, and *Ae. vexans* exhibited the most extensive geographic ranges across all scenarios, suggesting that these species should be studied more than the others under all scenarios. This is consistent with the observation that *Ae. albopictus* is included among the world’s most invasive species, highlighting its significant potential for risk and invasiveness [12,77,78]. Our study also indicates that *Ae. Epactius*, *Ae. Excrucians*, and *Ae. fitchii* appeared most frequently in the rankings of the top five range ratio indices, suggesting that they deserve increasing attention compared with other *Aedes* species. The lowest range similarity indices, i.e., the largest shifts in range position (centroids), were detected in *Ae. fitchii* and *Ae. epactius*, suggesting that a more considerable shift in priority regions should be implemented to combat their impacts under current–future scenarios compared with others.

Our study shows that the substantial increase in the OIHS of the 29 *Aedes* mosquito species under most future scenarios was mainly detected in North America, Europe, and Africa except desert regions, Eastern China, and tropical regions in Asia and the southeastern coastline regions of Australia, suggesting that these regions could be subjected to higher threats from *Aedes* mosquitoes under future compared to current scenarios. These regions were also the main bodies of the regions where the substantial increases in range overlap were identified under most of the future scenarios, suggesting that higher range overlaps of *Aedes* mosquito species could be detected in the future and necessitate stricter strategies to combat their impacts. Therefore, these regions should be a focus to address the effects of *Aedes* mosquitoes in future scenarios on human health and livestock breeding. Many studies have detected inter-specific competition among *Aedes* species, which could substantially mitigate their impacts on human, domestic, and farm animal health [79,80,81]. However, we did not include competition in our models. Strong inter-specific competition could occur in regions where substantial range overlaps and high OIHS were detected. This suggests that their impacts in these regions might be mitigated by inter-specific competition. Therefore, caution is needed when interpreting our observations.

## 5. Conclusions

Here, we present the first unified scheme on the global ranges and range overlap shifts in 29 *Aedes* mosquito species in the future. We detected expanded ranges in most of the *Aedes* mosquito species, and substantial increases in the index of habitat suitability overlap were detected in more than 70% of the global terrestrial area, suggesting an increasing impact on human health and livestock breeding in the future. Climatic predictors played stronger roles in their range expansions than other factors, suggesting that mitigating future climate change is one of the key approaches to combatting the impacts. We also identified the hotspots of overlapping ranges and substantial increases in range overlap in North America, Europe, and Africa, necessitating stricter strategies to mitigate their influences in the future. Our results indicate a globally increasing threat of *Aedes*-borne epidemic transmission in the future, suggesting that far more stringent management measures are needed.

## Data Availability

The original contributions presented in this study are included in the article/Supplementary Materials. Further inquiries can be directed to the corresponding authors.

## Supplementary Materials

The following supporting information can be downloaded at https://www.mdpi.com/article/10.3390/insects16050476/s1: Figure S1: Habitat suitability of the 29 *Aedes* mosquitoes; Figure S2: Ranges of the 29 *Aedes* mosquitoes; Table S1: Importance of 30 variables in the full models; Table S2: Correlation between 30 predictors; Table S3: Algorithms in baseline models; Table S4: Model performance; Table S5: Predictors’ importance values in baseline SDMs; Table S6: MSS thresholds used to define potential ranges.
