# Supplementary material for: Future Climate Predicts Range Shifts and Increased Global Habitat Suitability for 29 Aedes Mosquito Species
Source: Insects. 2025 Apr 30;16(5):476. doi: 10.3390/insects16050476 (PMC12111898; doi:10.3390/insects16050476)
Supplement: Supplementary file 1 [file insects-16-00476-s001.zip › Supporting files/Figure S1 Habitat suitability of the 29 major Aedes mosquitoes.pdf]

Figure S1 Habitat suitability of the 29 major *Aedes* mosquitoes

Aedes aegypti

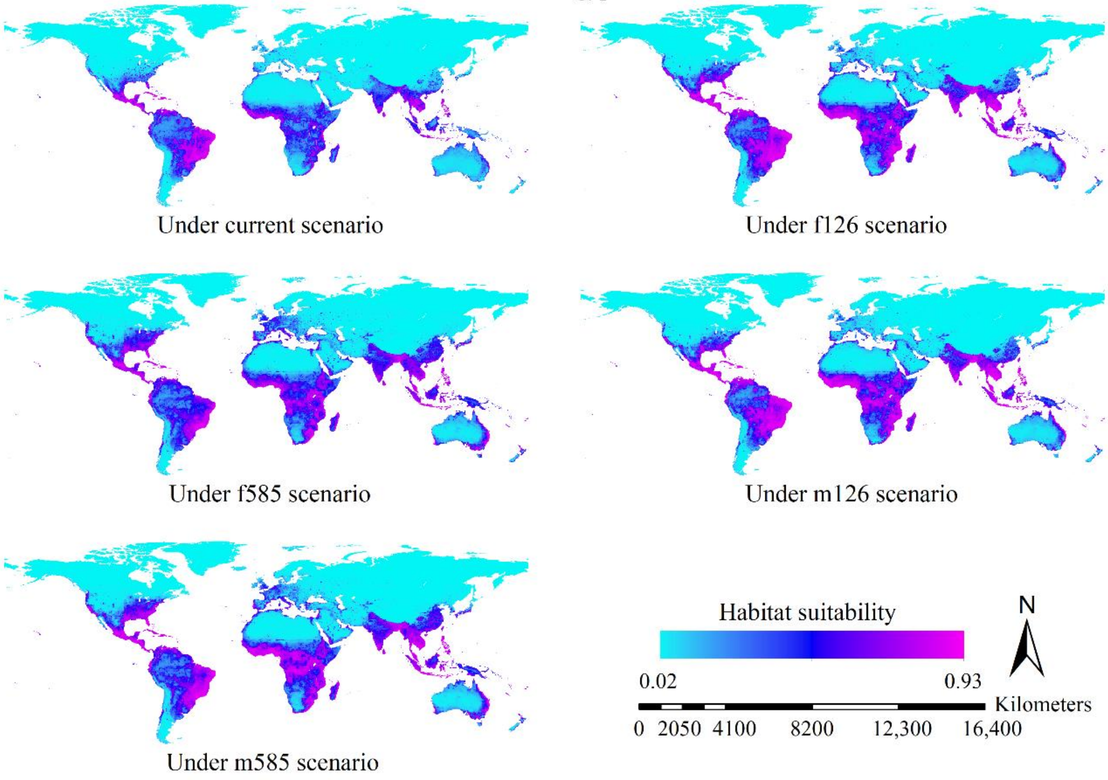

Aedes albopictus

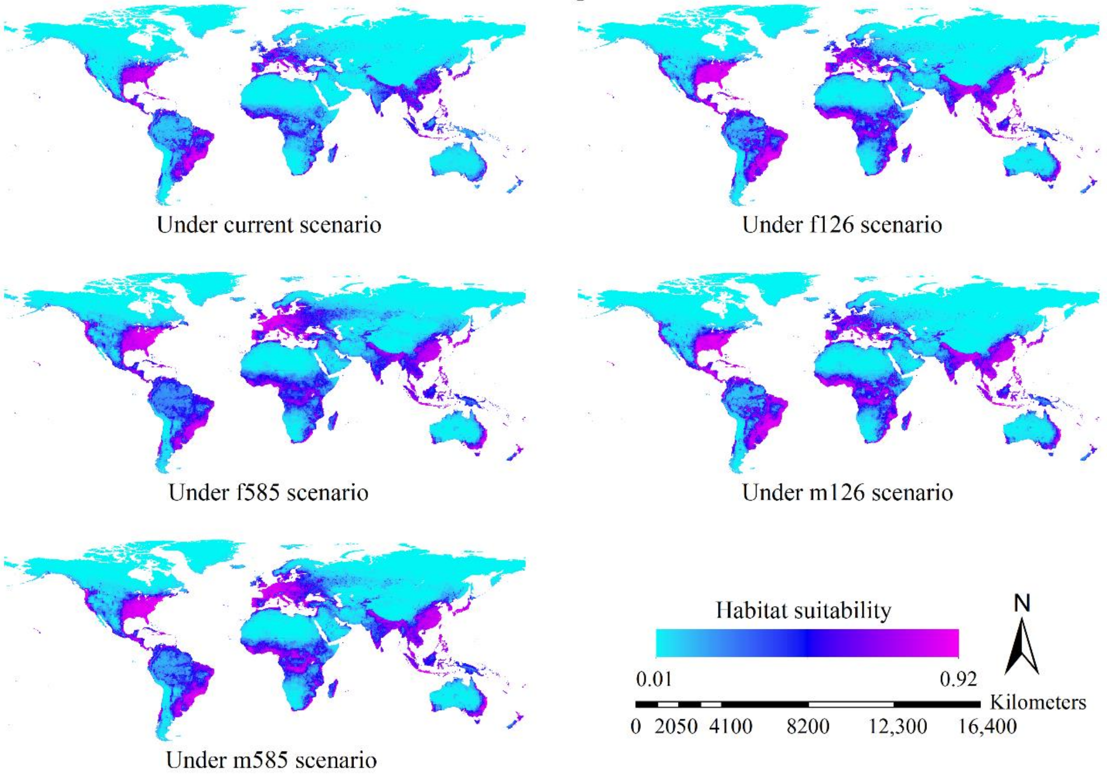

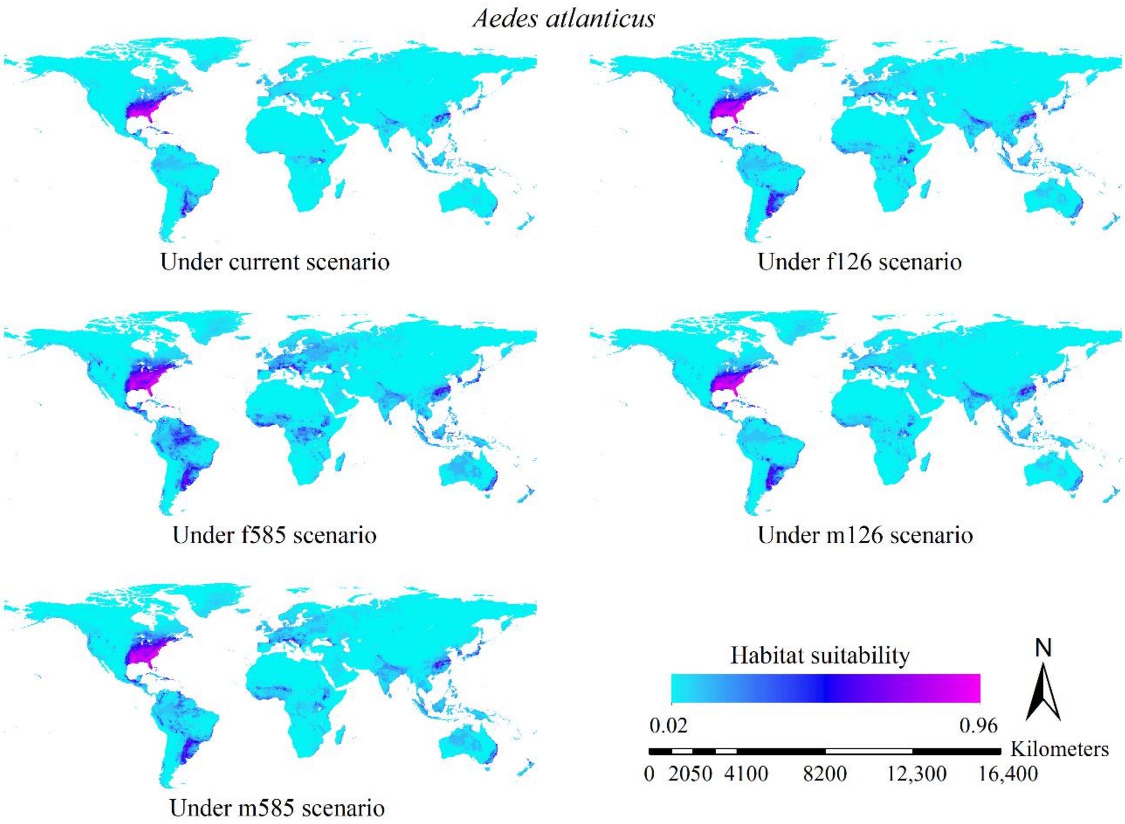
Aedes atlanticus
Under current scenario
Under f126 scenario
Under f585 scenario
Under m126 scenario
Under m585 scenario
Habitat suitability
0.02
0.96
N
Kilometers
0 2050 4100 8200 12,300 16,400

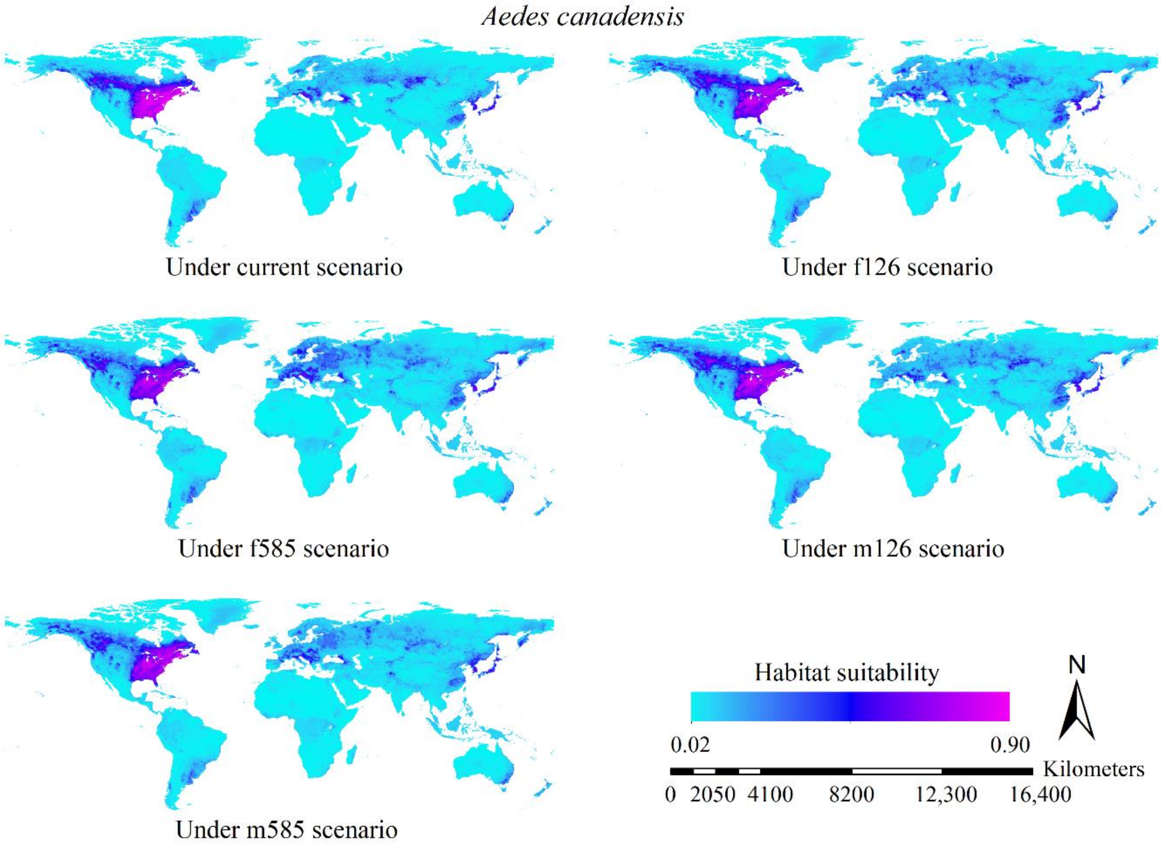
Aedes canadensis
Under current scenario
Under f126 scenario
Under f585 scenario
Under m126 scenario
Under m585 scenario
Habitat suitability
0.02
0.90
N
Kilometers
0 2050 4100 8200 12,300 16,400

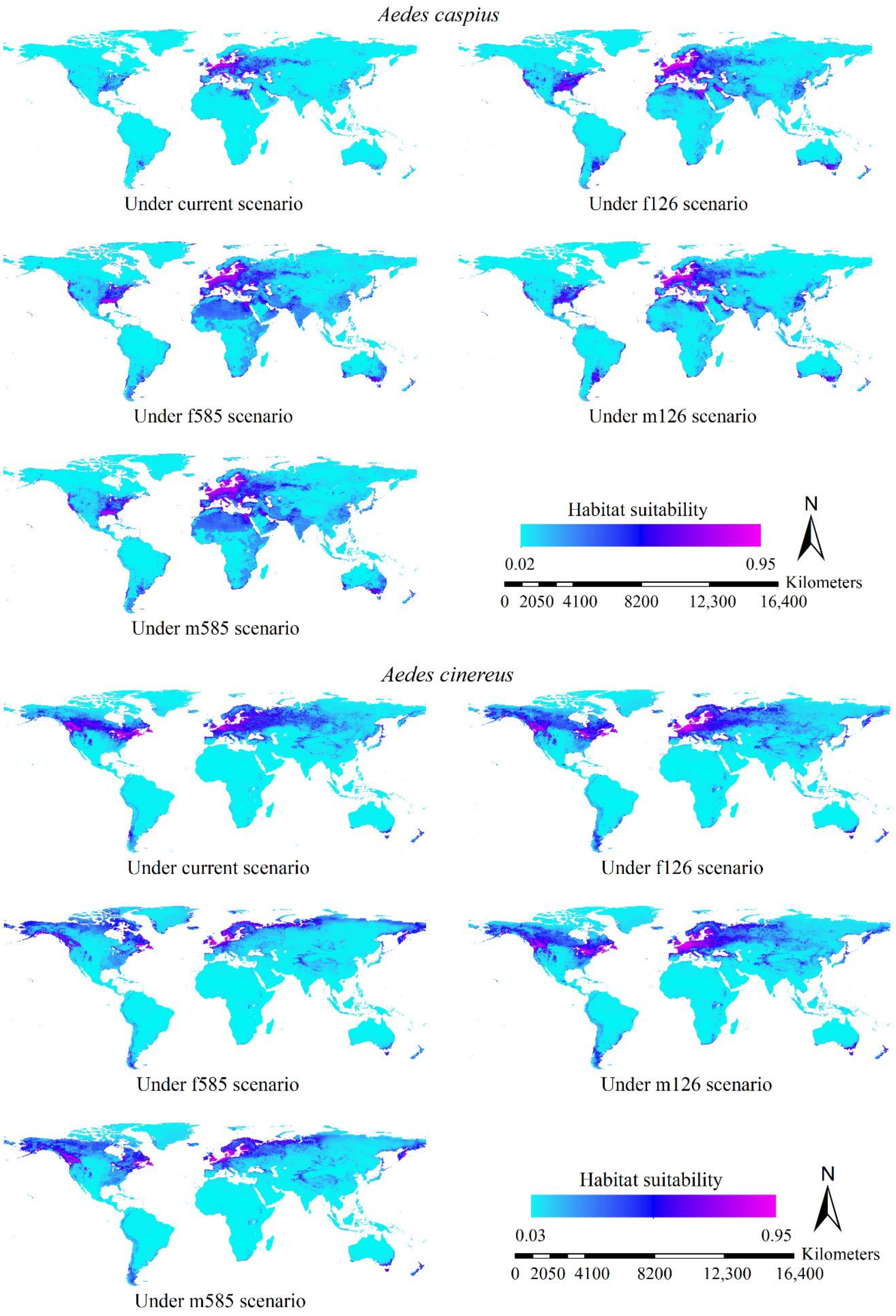
Aedes caspius
Under current scenario
Under f126 scenario
Under f585 scenario
Under m126 scenario
Under m585 scenario
Habitat suitability
0.02
0.95
N
Kilometers
0 2050 4100 8200 12,300 16,400
Aedes cinereus
Under current scenario
Under f126 scenario
Under f585 scenario
Under m126 scenario
Under m585 scenario
Habitat suitability
0.03
0.95
N
Kilometers
0 2050 4100 8200 12,300 16,400

Aedes communis

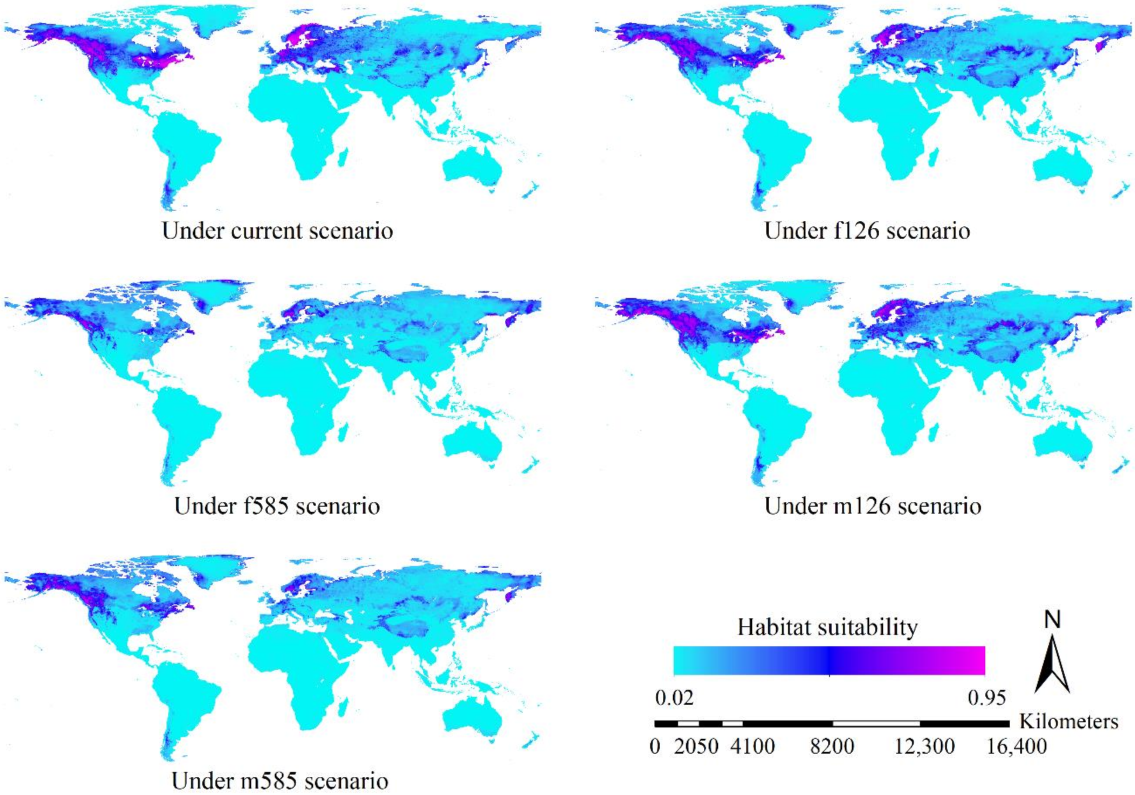

Aedes dorsalis

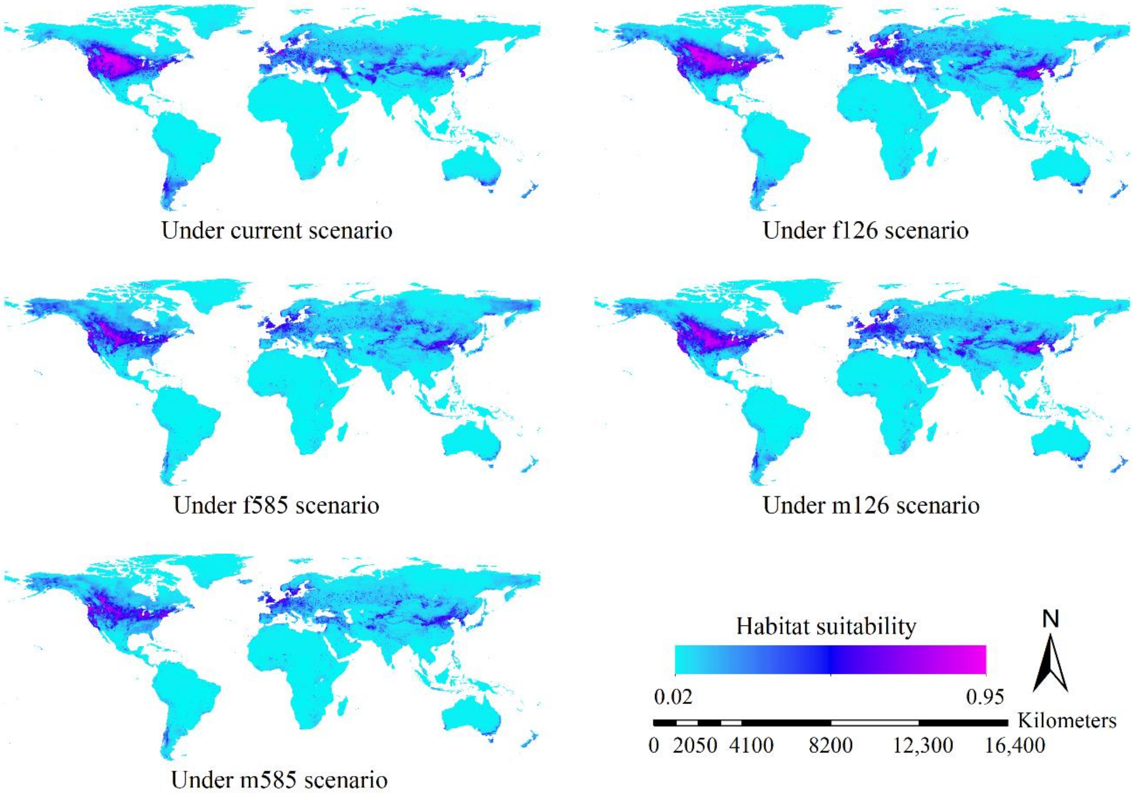

Aedes epactius

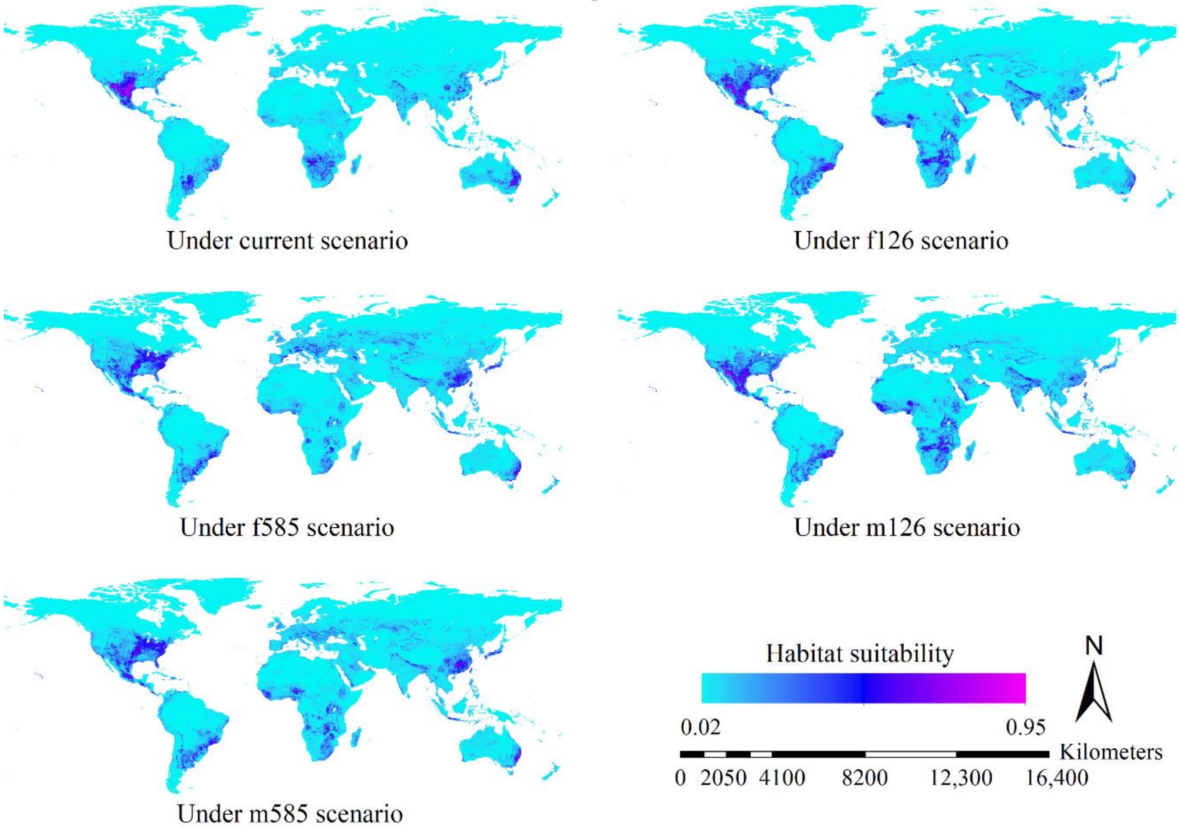

Aedes excrucians

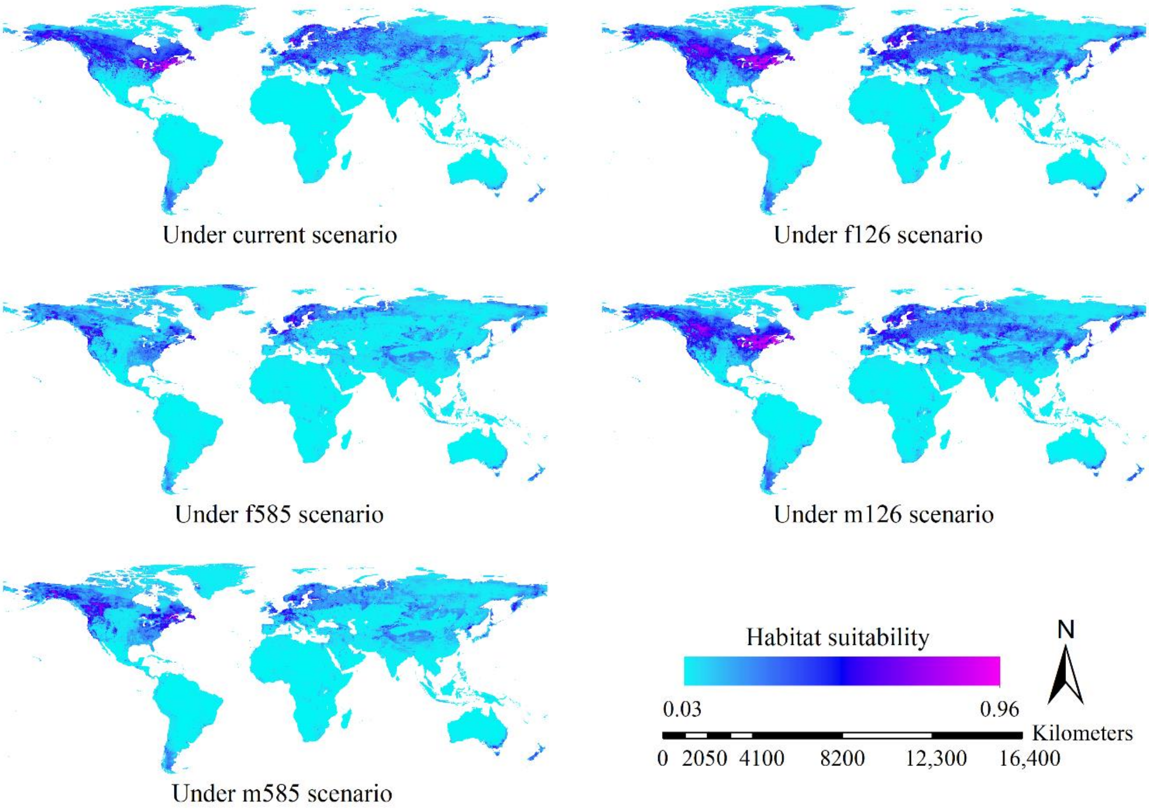

Aedes fitchii

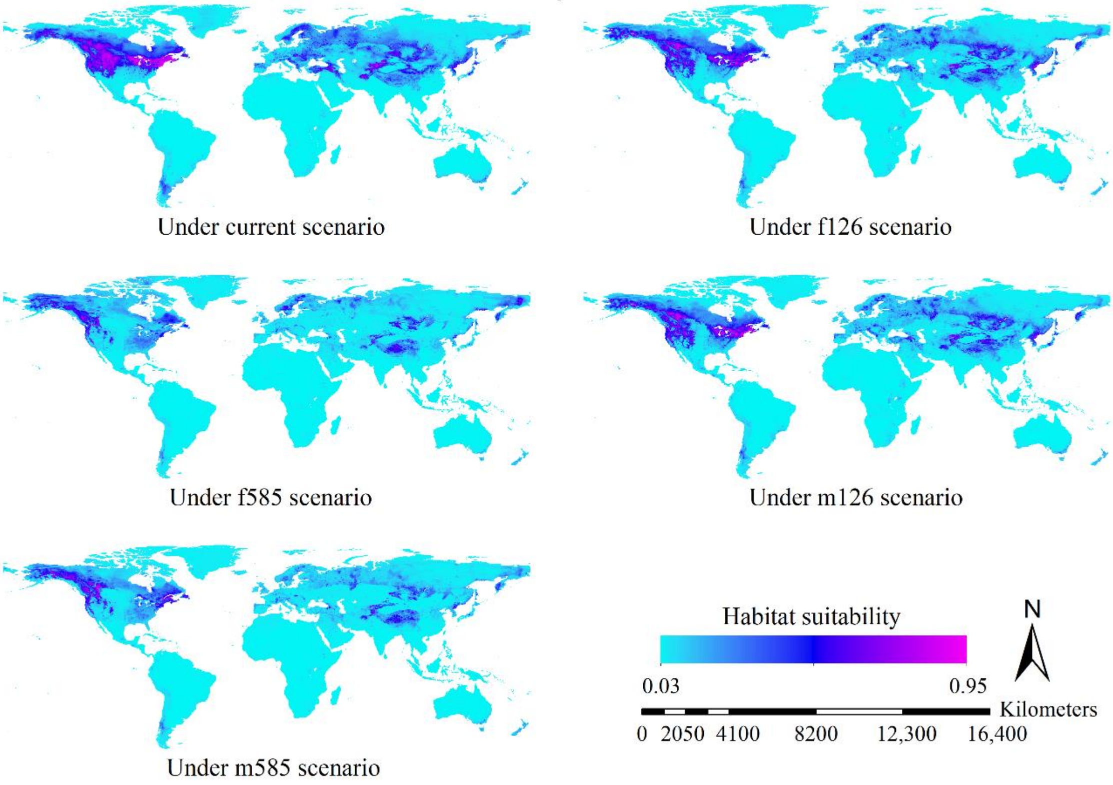

Aedes geniculatus

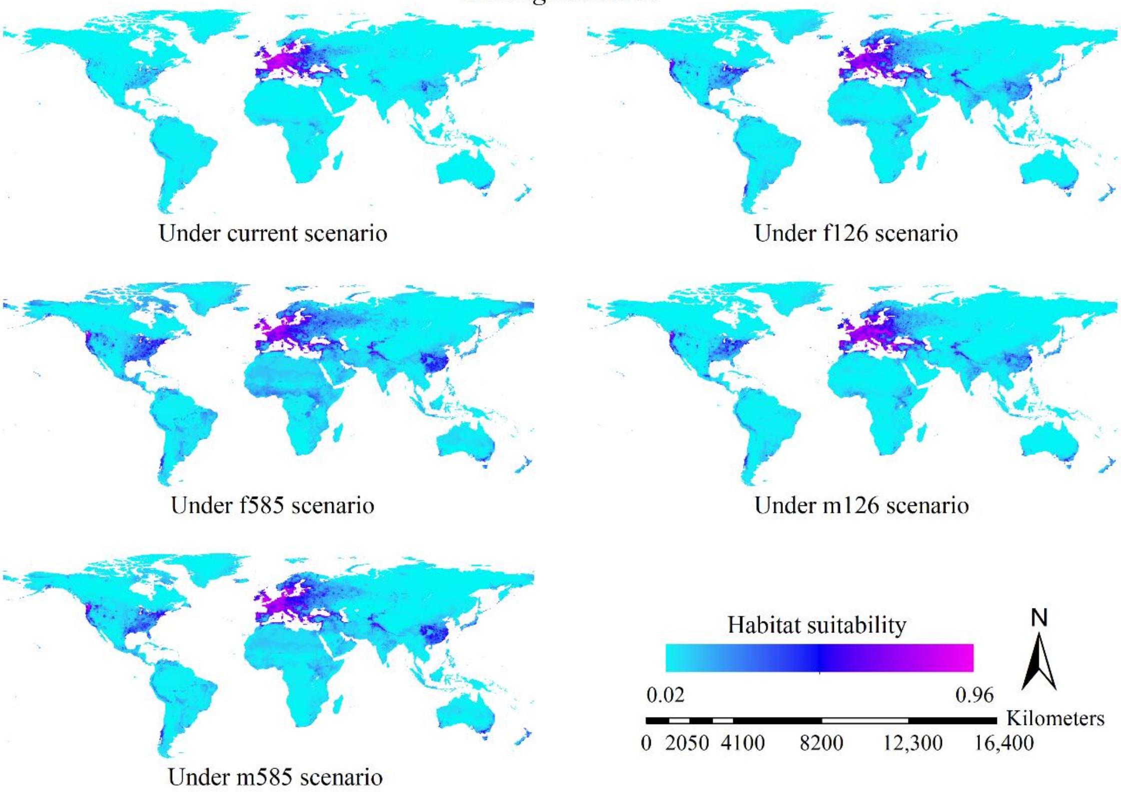

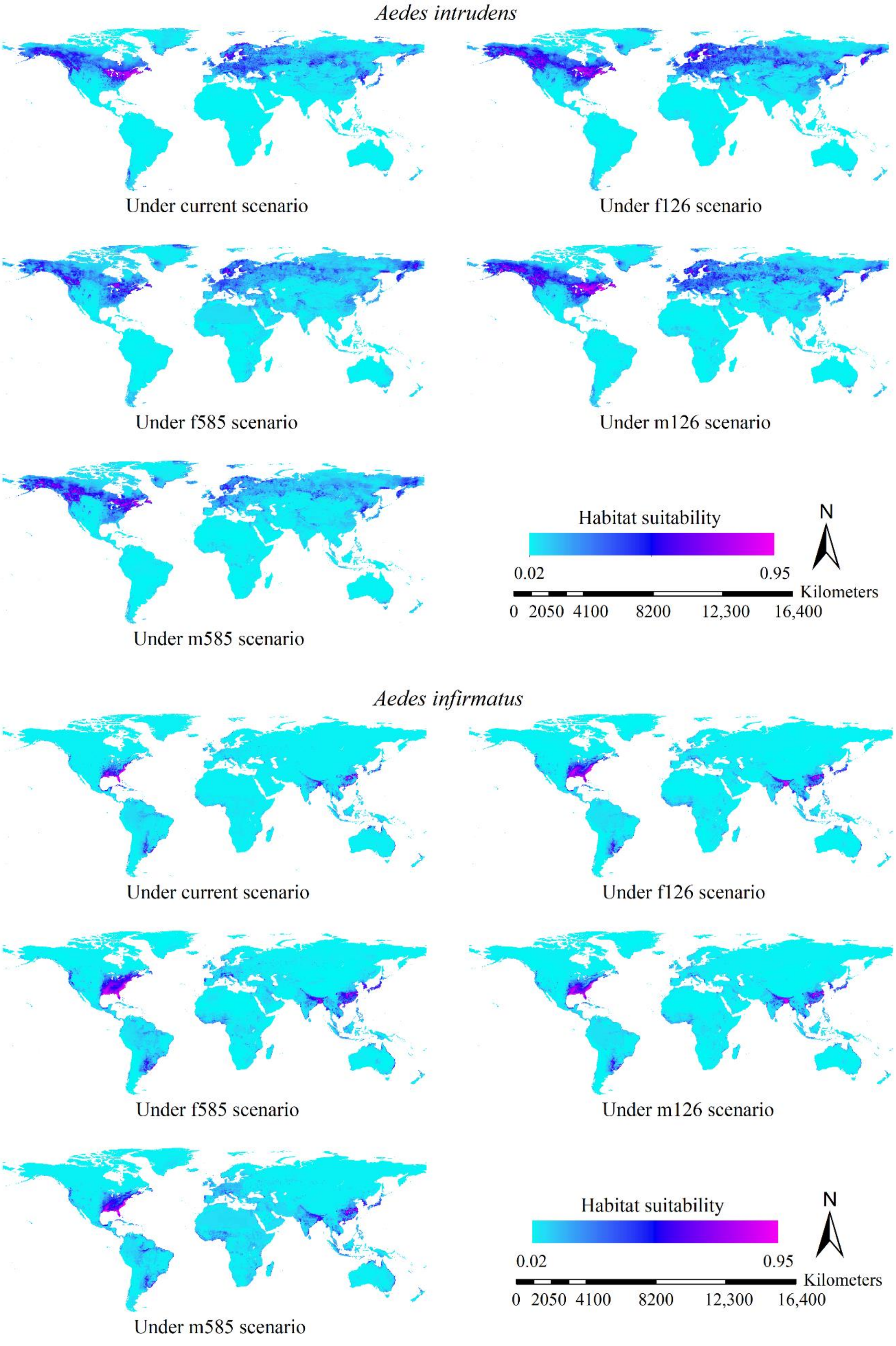

Aedes intrudens
Under current scenario
Under f126 scenario
Under f585 scenario
Under m126 scenario
Under m585 scenario
Habitat suitability
0.02
0.95
N
Kilometers
0 2050 4100 8200 12,300 16,400
Aedes infirmatus
Under current scenario
Under f126 scenario
Under f585 scenario
Under m126 scenario
Under m585 scenario
Habitat suitability
0.02
0.95
N
Kilometers
0 2050 4100 8200 12,300 16,400

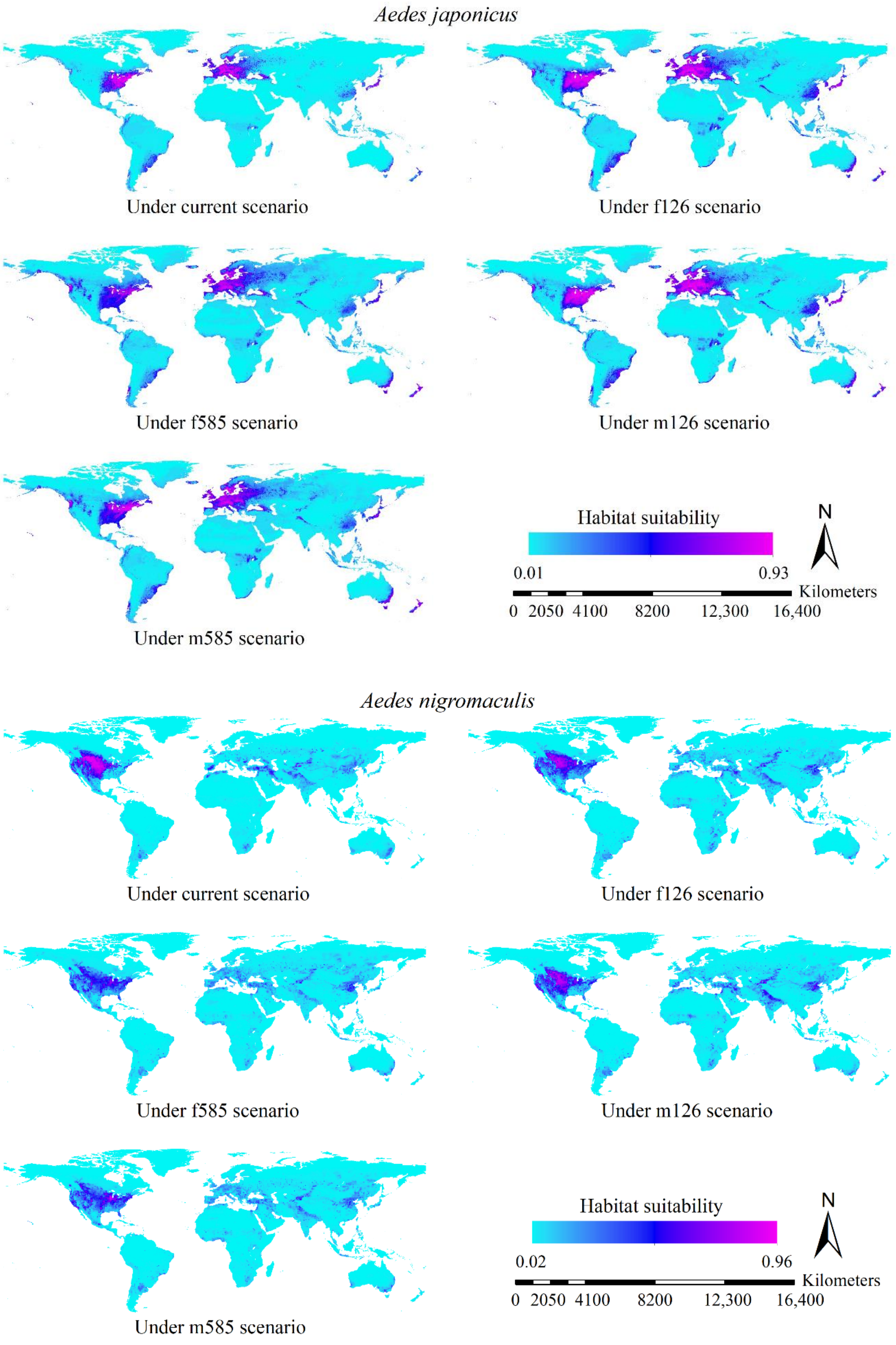

Aedes japonicus
Under current scenario
Under f126 scenario
Under f585 scenario
Under m126 scenario
Under m585 scenario
Habitat suitability
0.01
0.93
N
Kilometers
0 2050 4100 8200 12,300 16,400
Aedes nigromaculis
Under current scenario
Under f126 scenario
Under f585 scenario
Under m126 scenario
Under m585 scenario
Habitat suitability
0.02
0.96
N
Kilometers
0 2050 4100 8200 12,300 16,400

Aedes notoscriptus

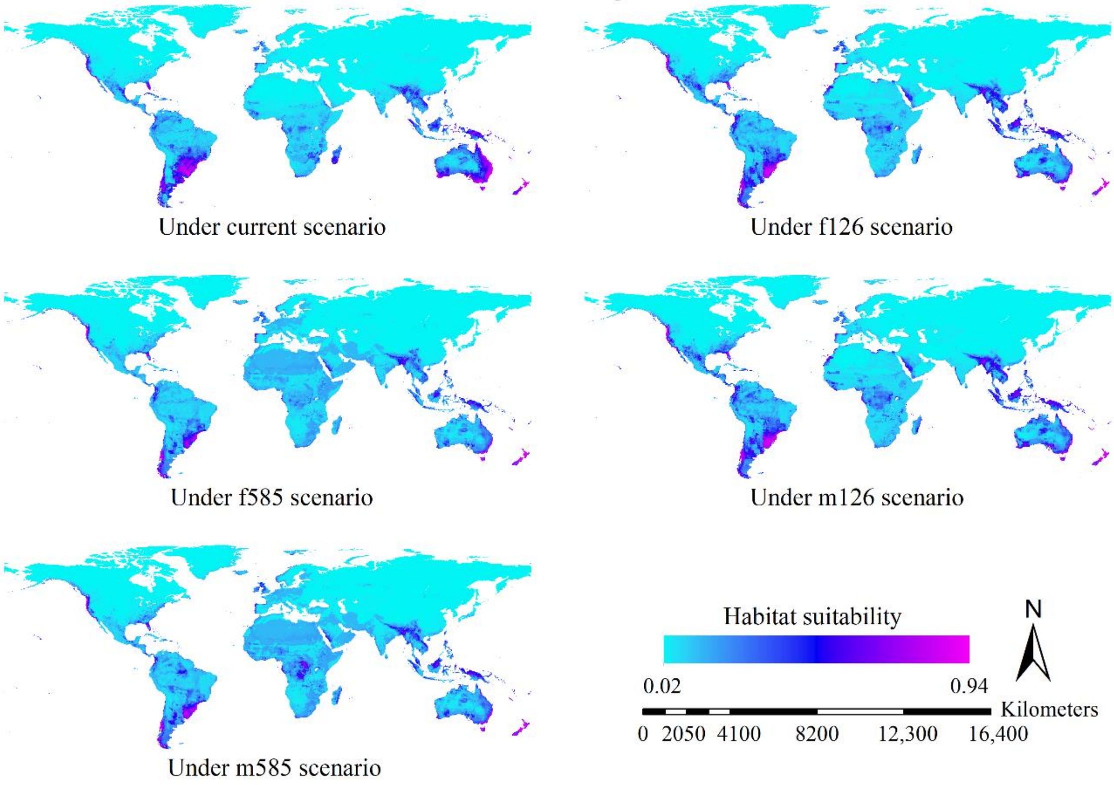

Aedes provocans

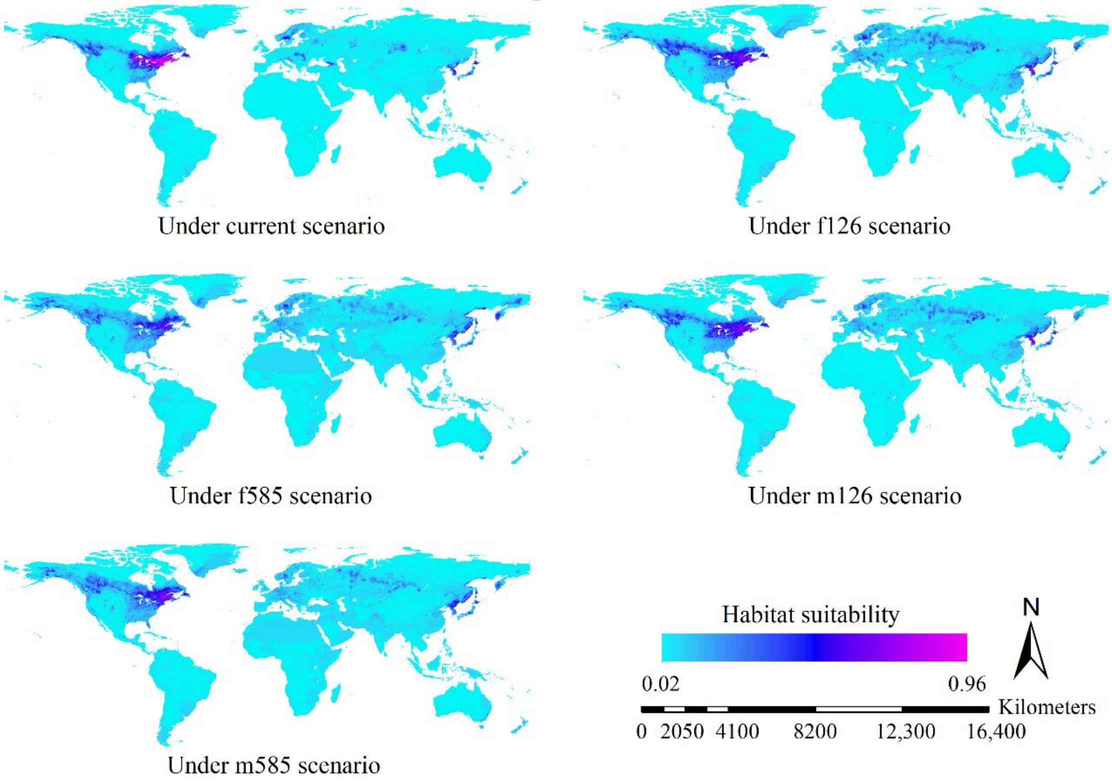

Aedes punctor

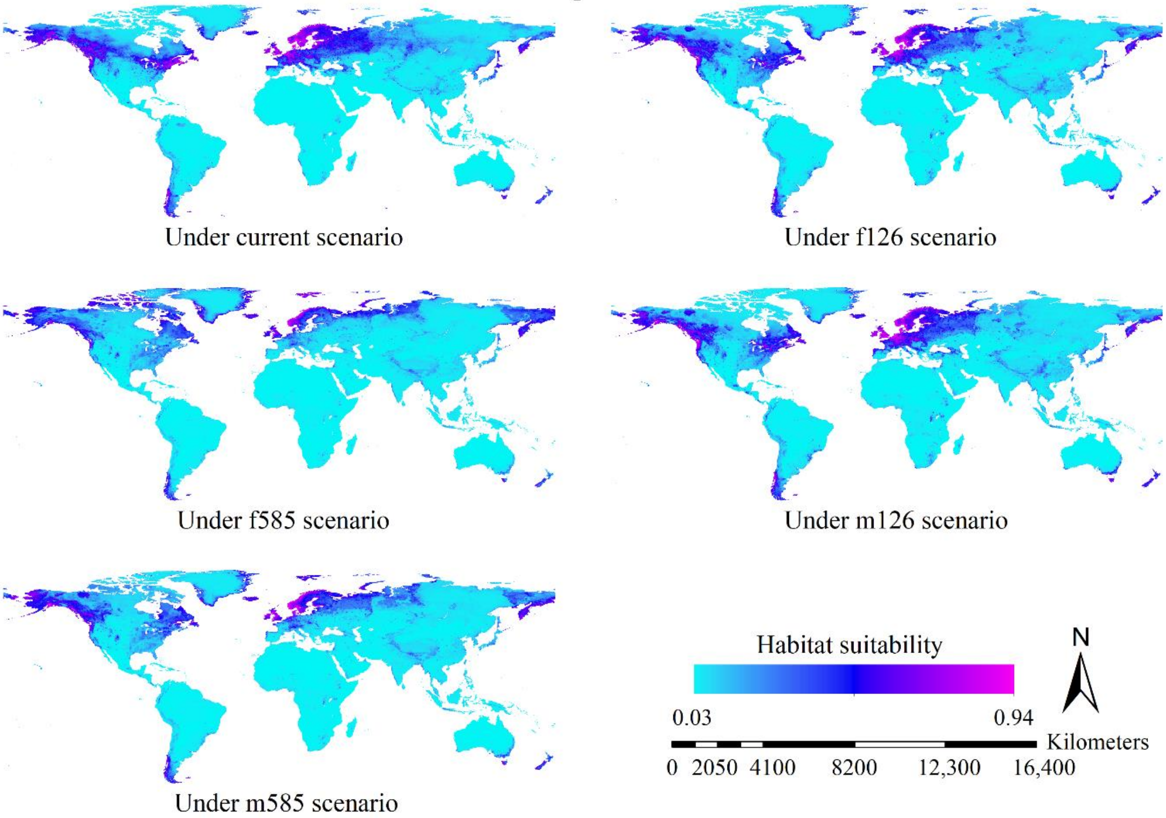

Aedes rubrithorax

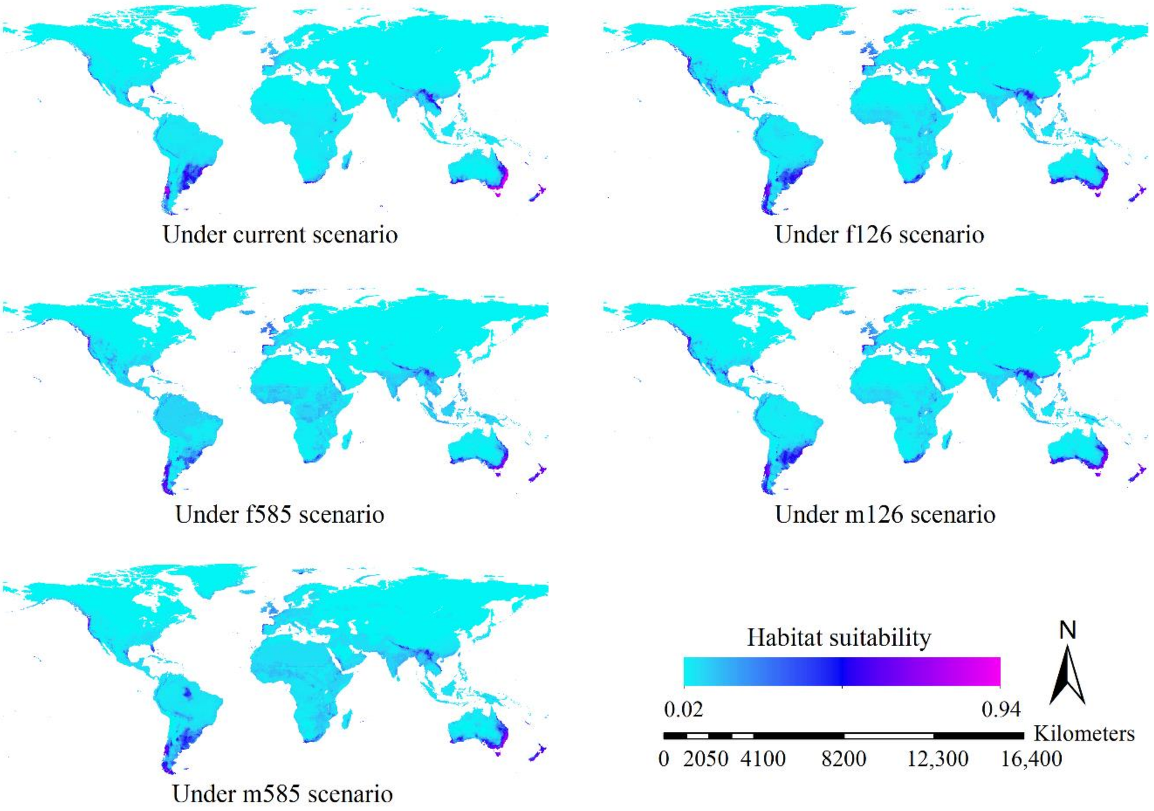

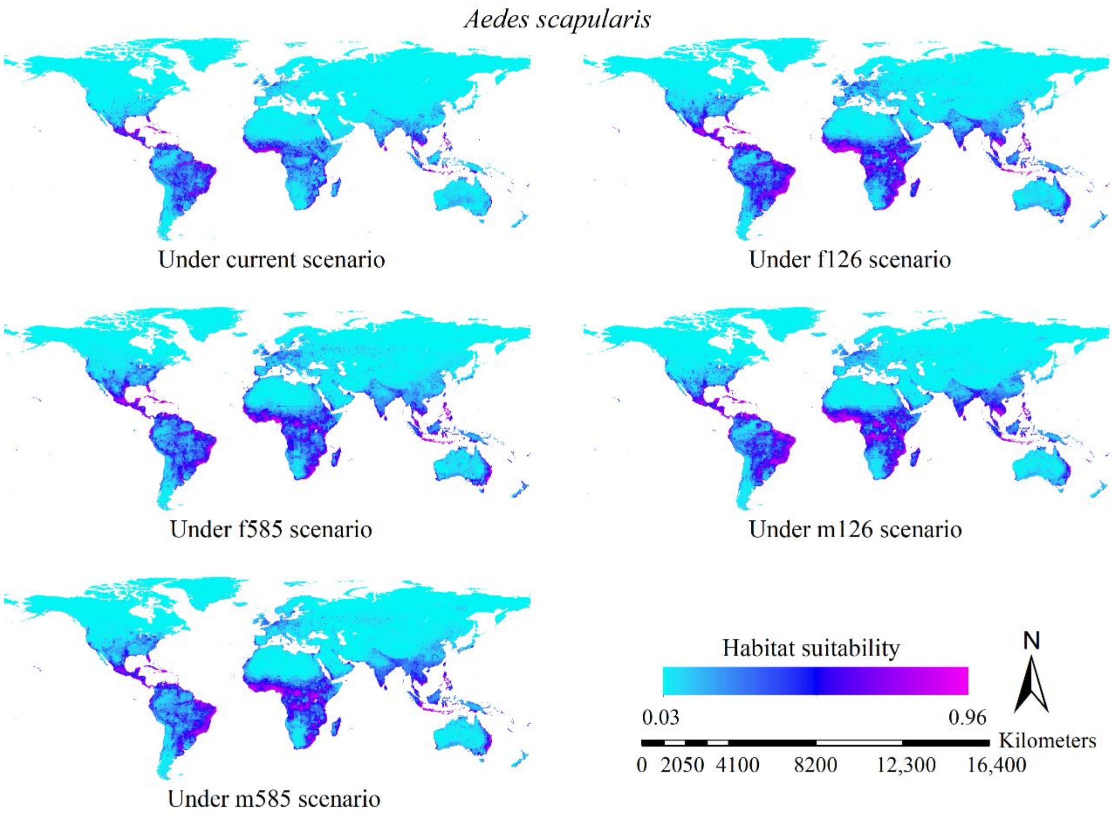
Aedes scapularis
Under current scenario
Under f126 scenario
Under f585 scenario
Under m126 scenario
Under m585 scenario
Habitat suitability
0.03
0.96
N
Kilometers
0 2050 4100 8200 12,300 16,400

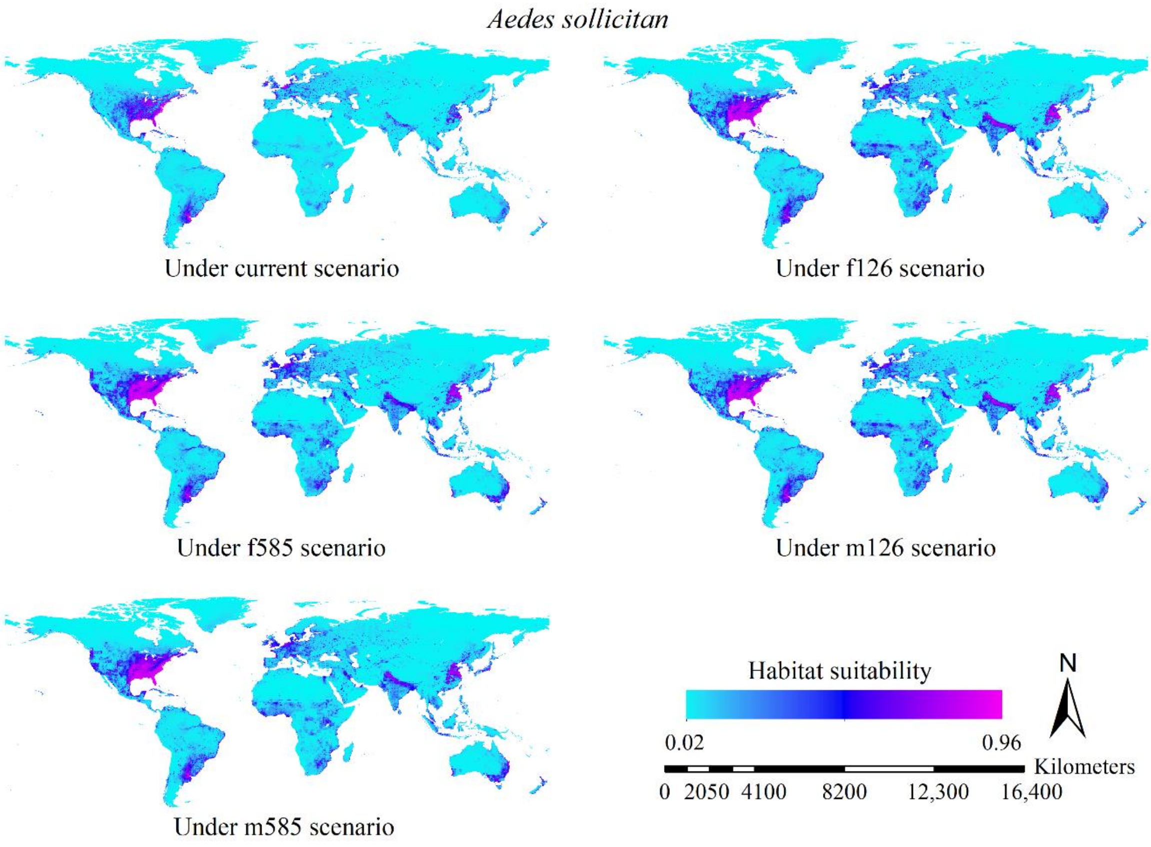
Aedes sollicitan
Under current scenario
Under f126 scenario
Under f585 scenario
Under m126 scenario
Under m585 scenario
Habitat suitability
0.02
0.96
N
Kilometers
0 2050 4100 8200 12,300 16,400

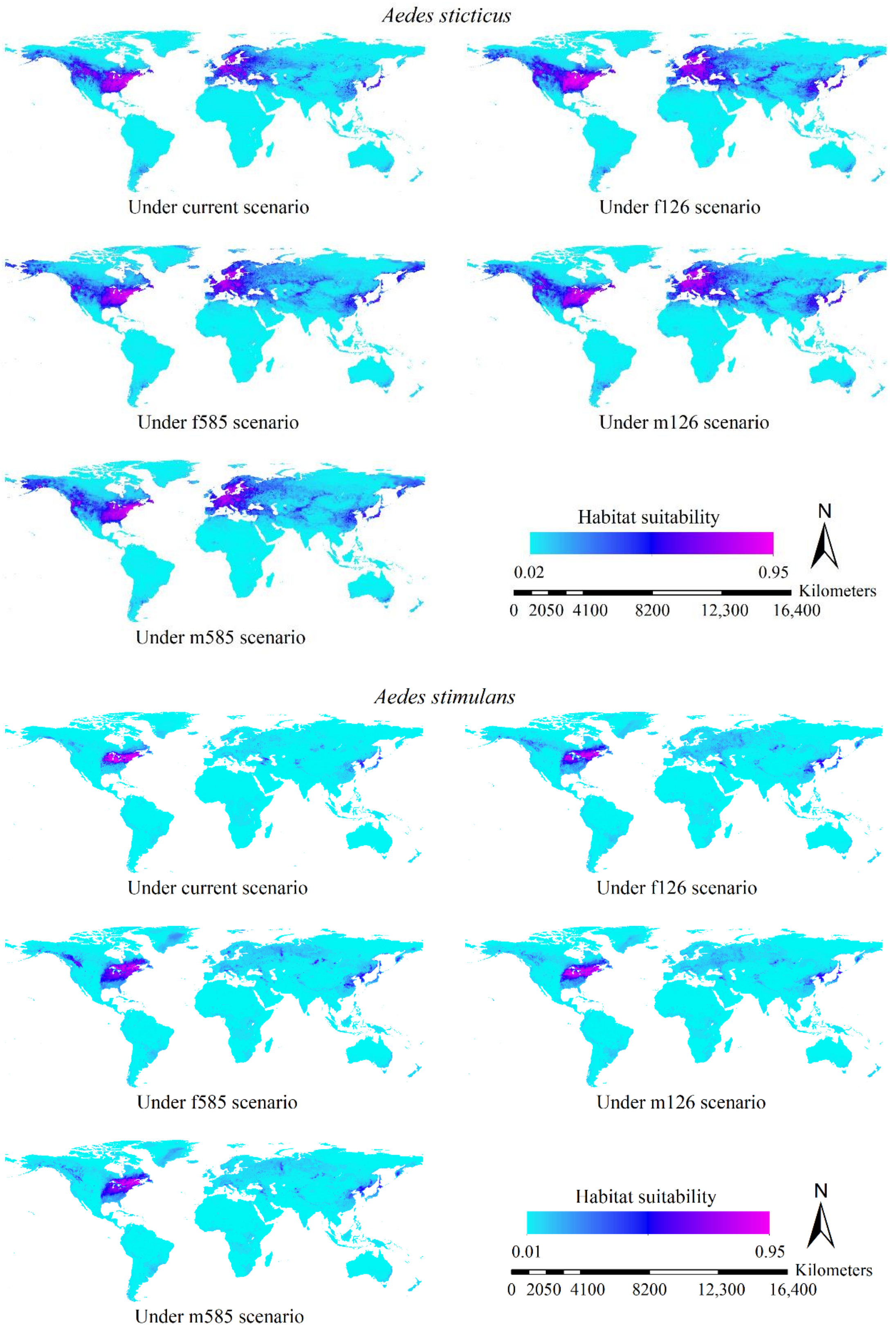

Aedes sticticus
Under current scenario
Under f126 scenario
Under f585 scenario
Under m126 scenario
Under m585 scenario
Habitat suitability
0.02
0.95
N
Kilometers
0 2050 4100 8200 12,300 16,400
Aedes stimulans
Under current scenario
Under f126 scenario
Under f585 scenario
Under m126 scenario
Under m585 scenario
Habitat suitability
0.01
0.95
N
Kilometers
0 2050 4100 8200 12,300 16,400

Aedes taeniorhynchus

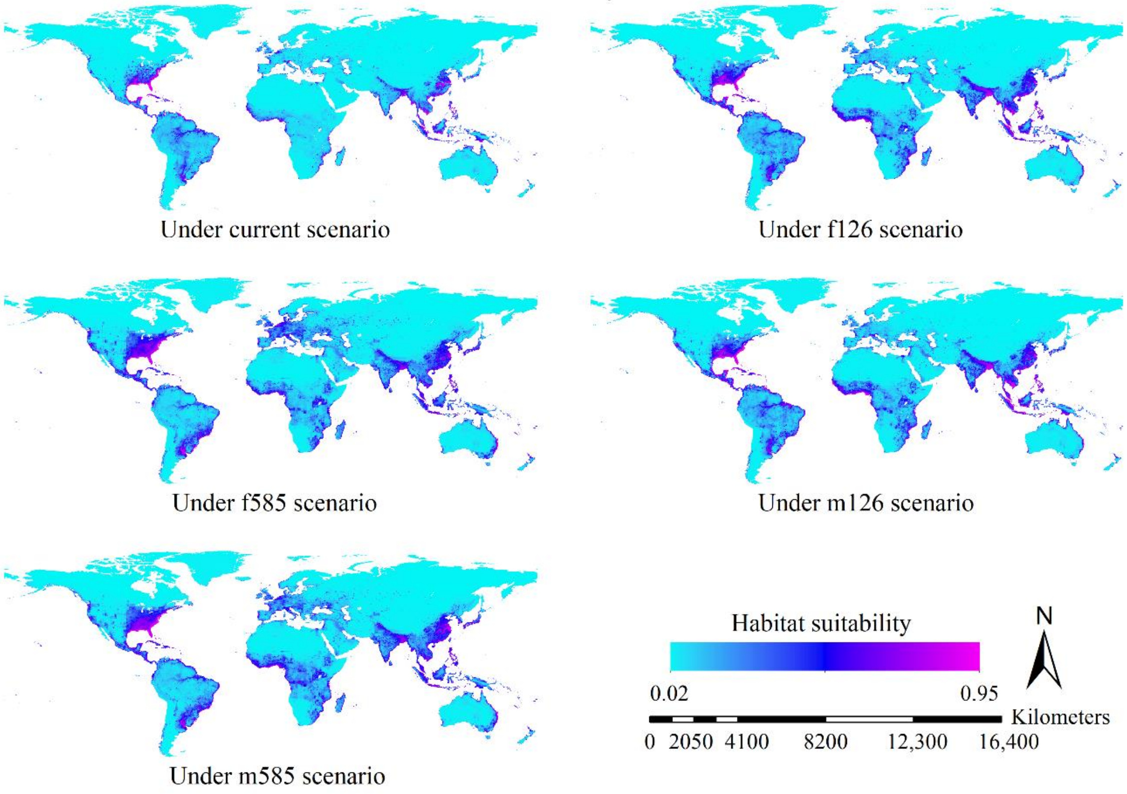

Aedes triseriatus

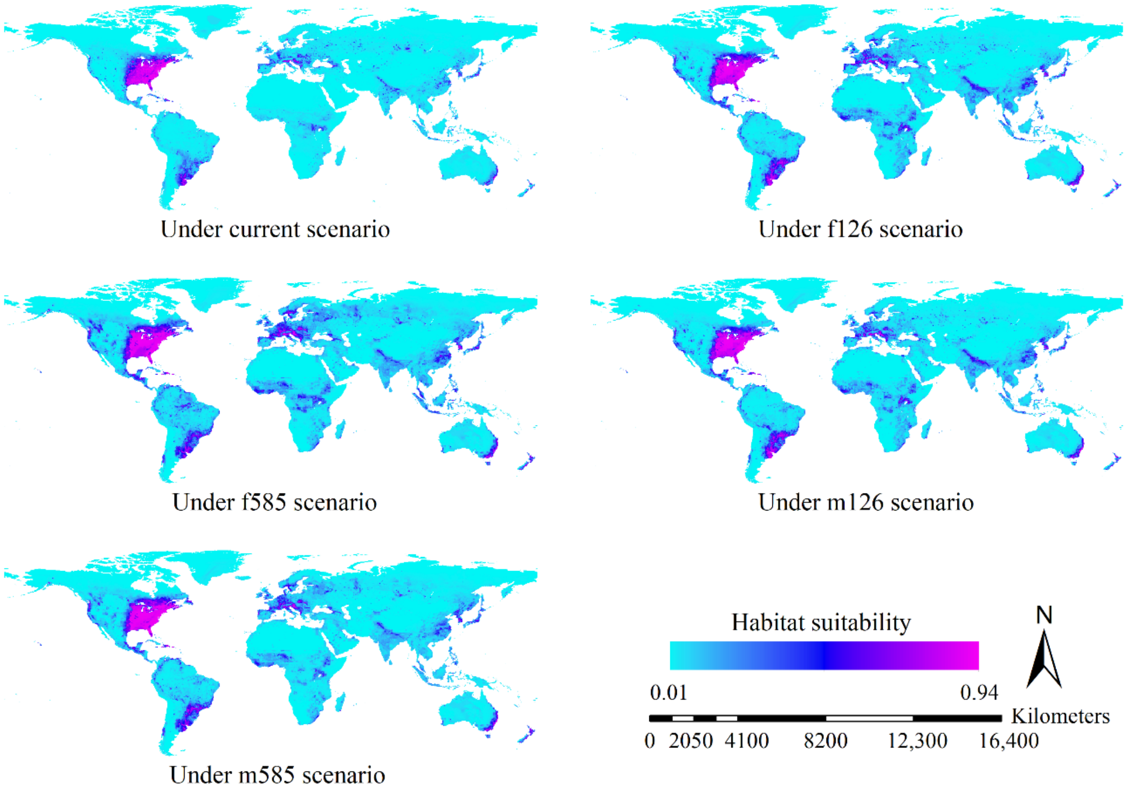

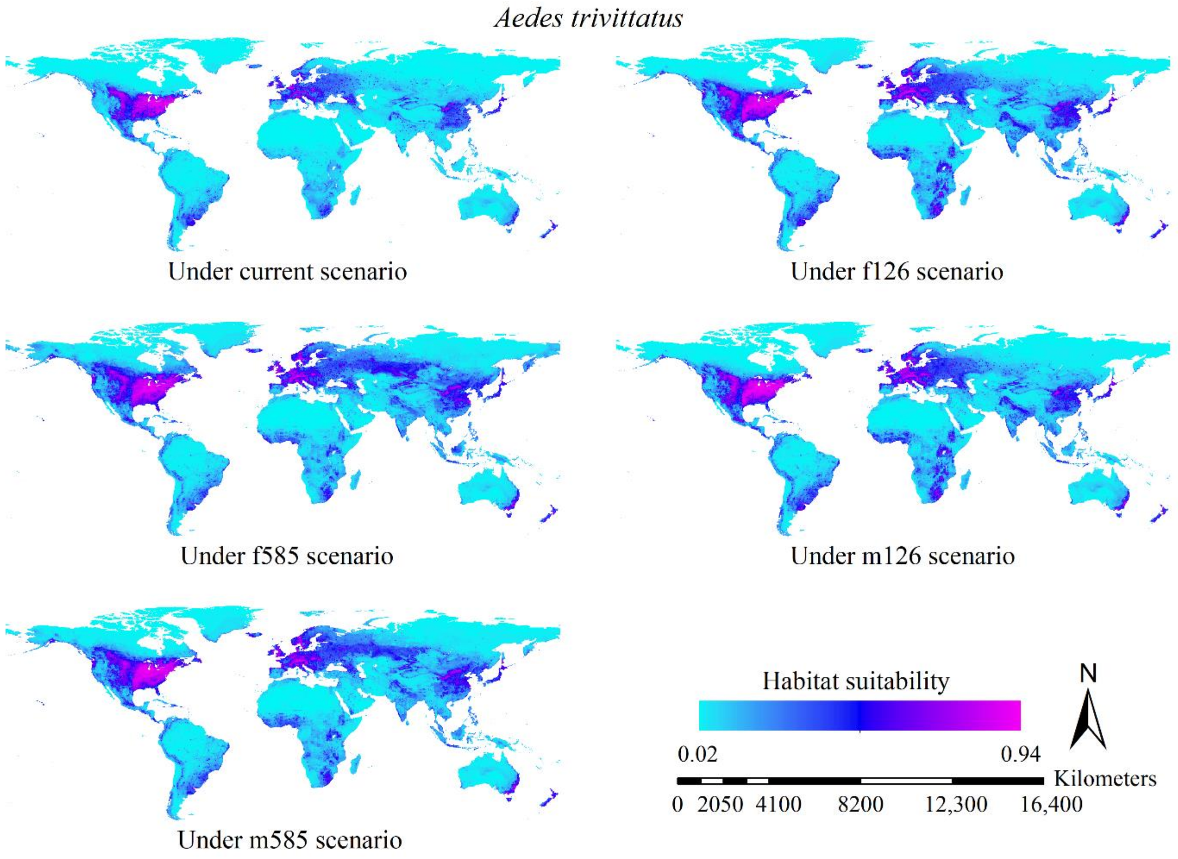
Aedes trivittatus
Under current scenario
Under f126 scenario
Under f585 scenario
Under m126 scenario
Under m585 scenario
Habitat suitability
0.02
0.94
N
Kilometers
0 2050 4100 8200 12,300 16,400

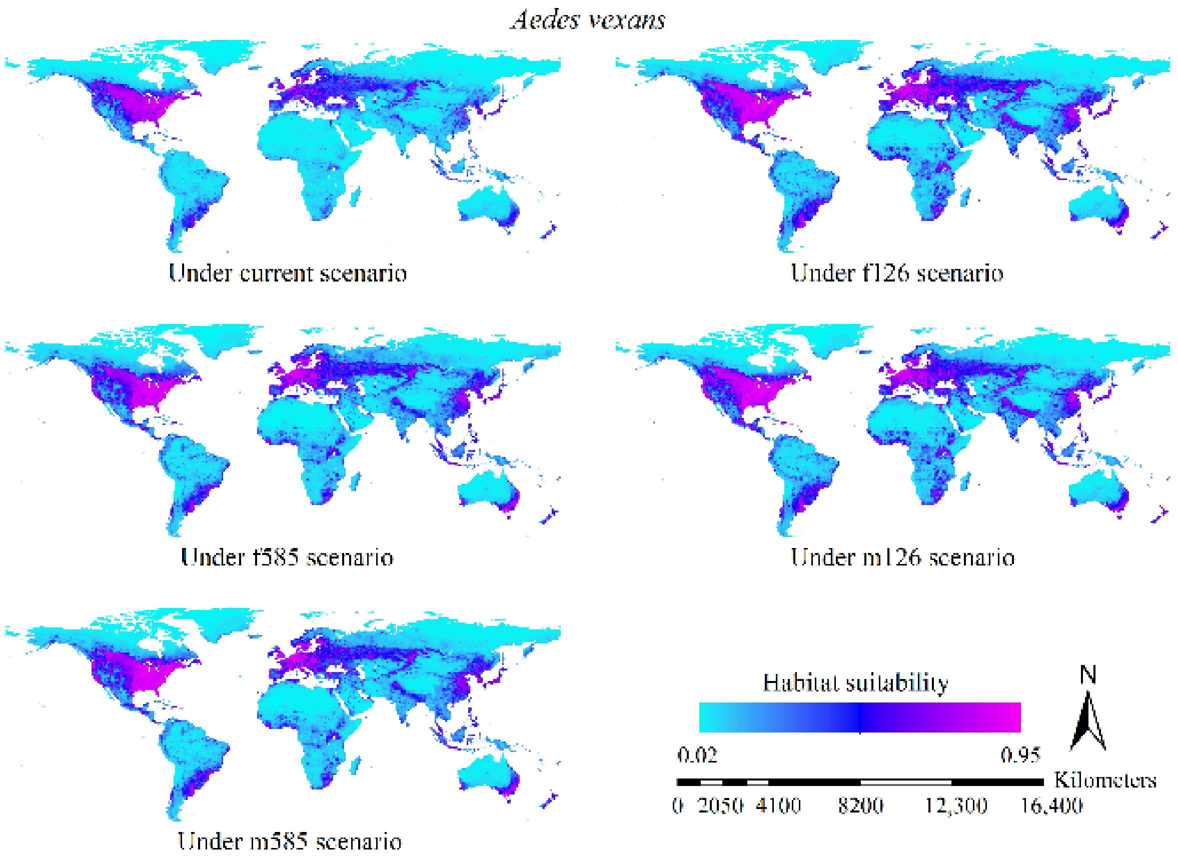
Aedes vexans
Under current scenario
Under f126 scenario
Under f585 scenario
Under m126 scenario
Under m585 scenario
Habitat suitability
0.02
0.95
N
Kilometers
0 2050 4100 8200 12,300 16,400

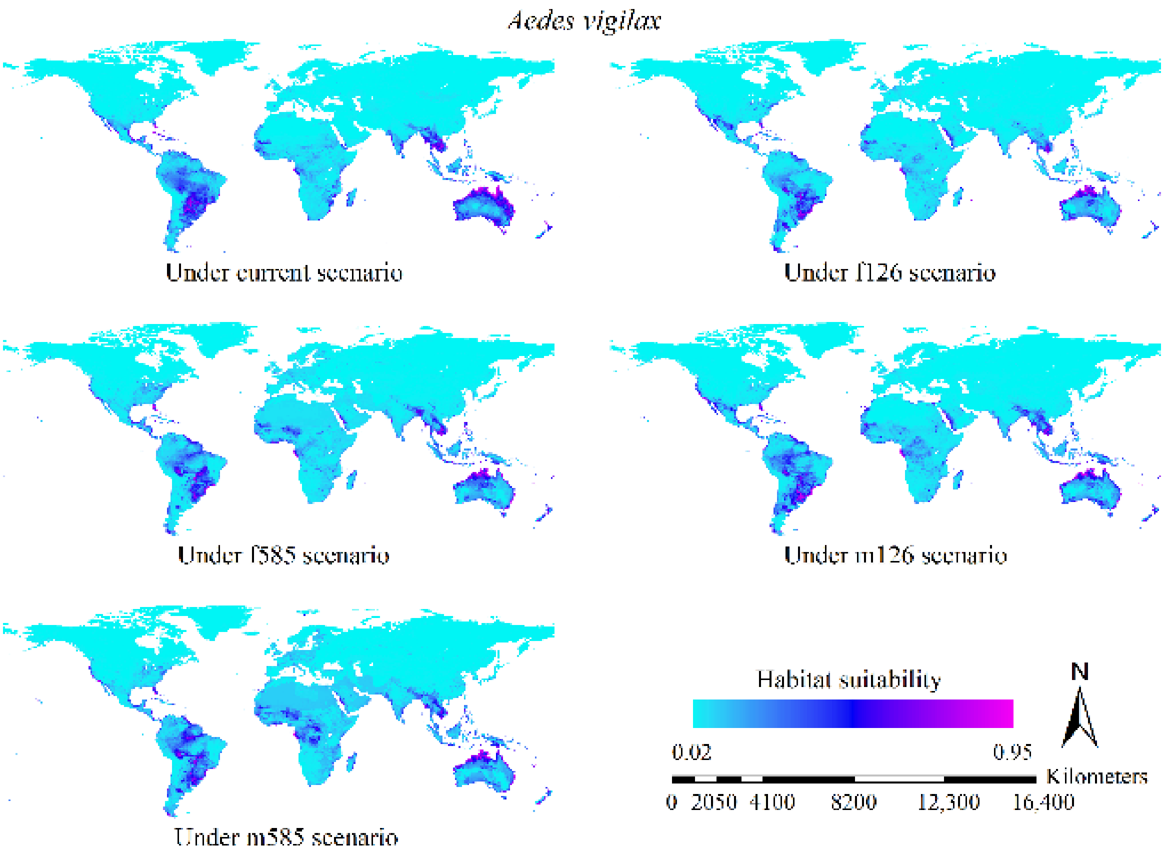

Aedes vigilax
Under current scenario
Under f126 scenario
Under f585 scenario
Under m126 scenario
Under m585 scenario
Habitat suitability
0.02
0.95
N
Kilometers
0 2050 4100 8200 12,300 16,400
