# Supplementary material for: Future Climate Predicts Range Shifts and Increased Global Habitat Suitability for 29 Aedes Mosquito Species
Source: Insects. 2025 Apr 30;16(5):476. doi: 10.3390/insects16050476 (PMC12111898; doi:10.3390/insects16050476)
Supplement: Supplementary file 1 [file insects-16-00476-s001.zip › Supporting files/Figure S2 Ranges of the 29 major Aedes mosquitoes.pdf]

Figure S2 Ranges of the 29 major *Aedes* mosquitoes

Aedes aegypti

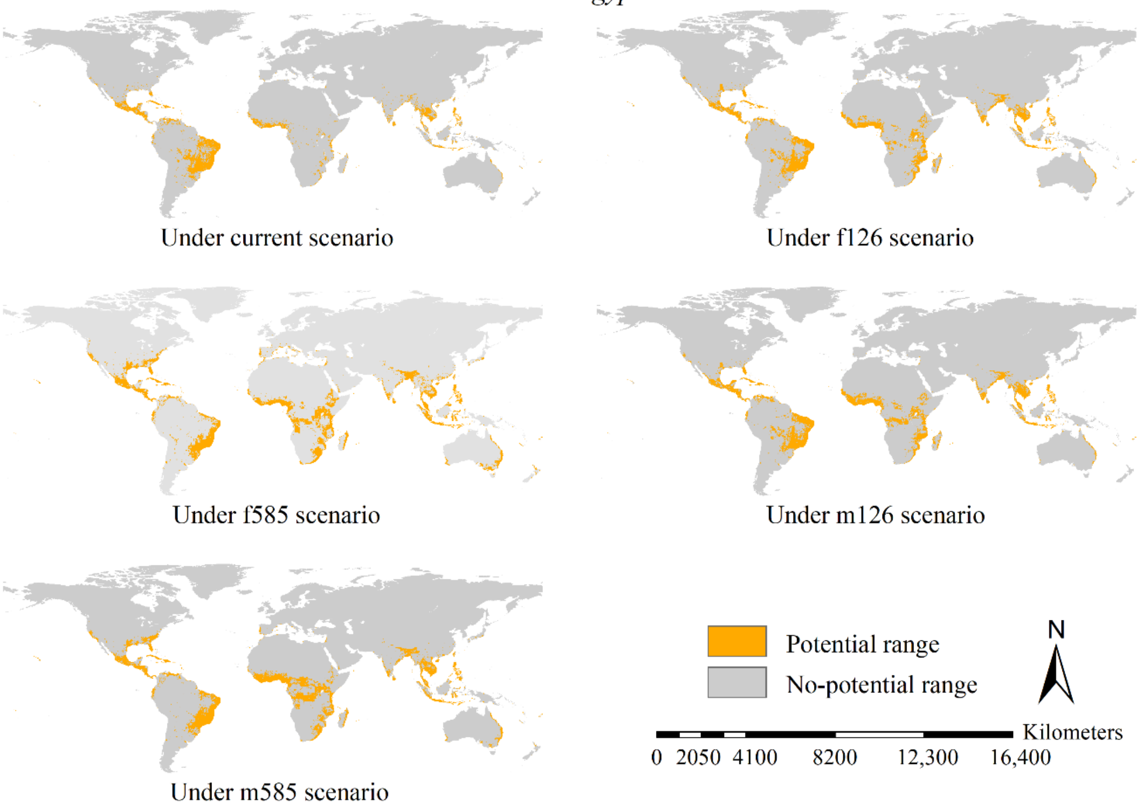

Aedes albopictus

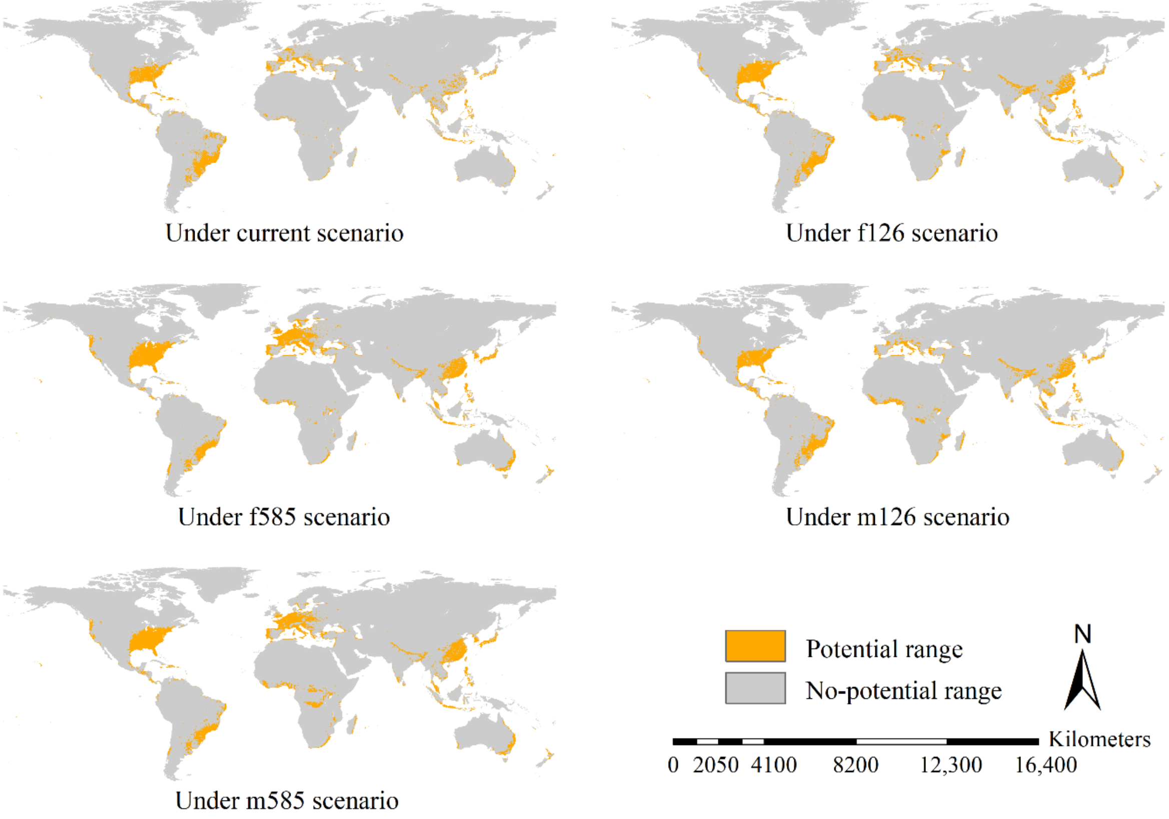

Aedes atlanticus

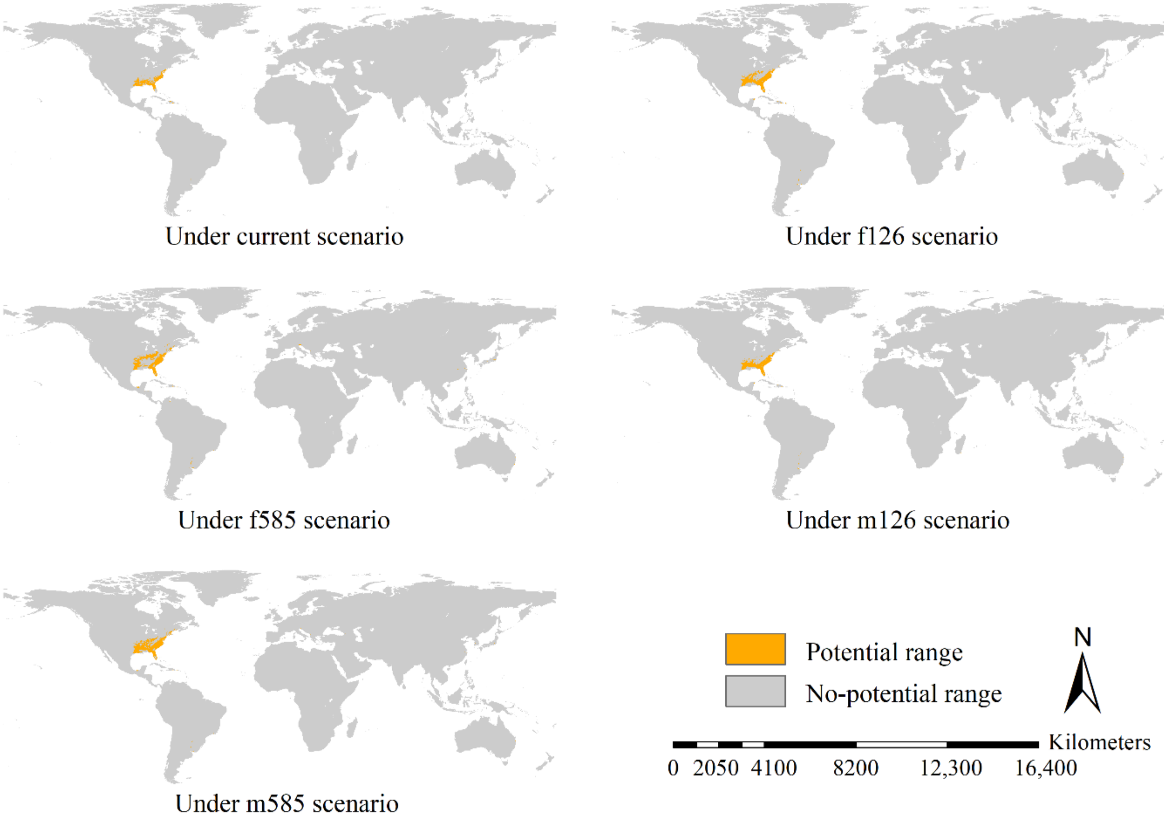

Aedes canadensis

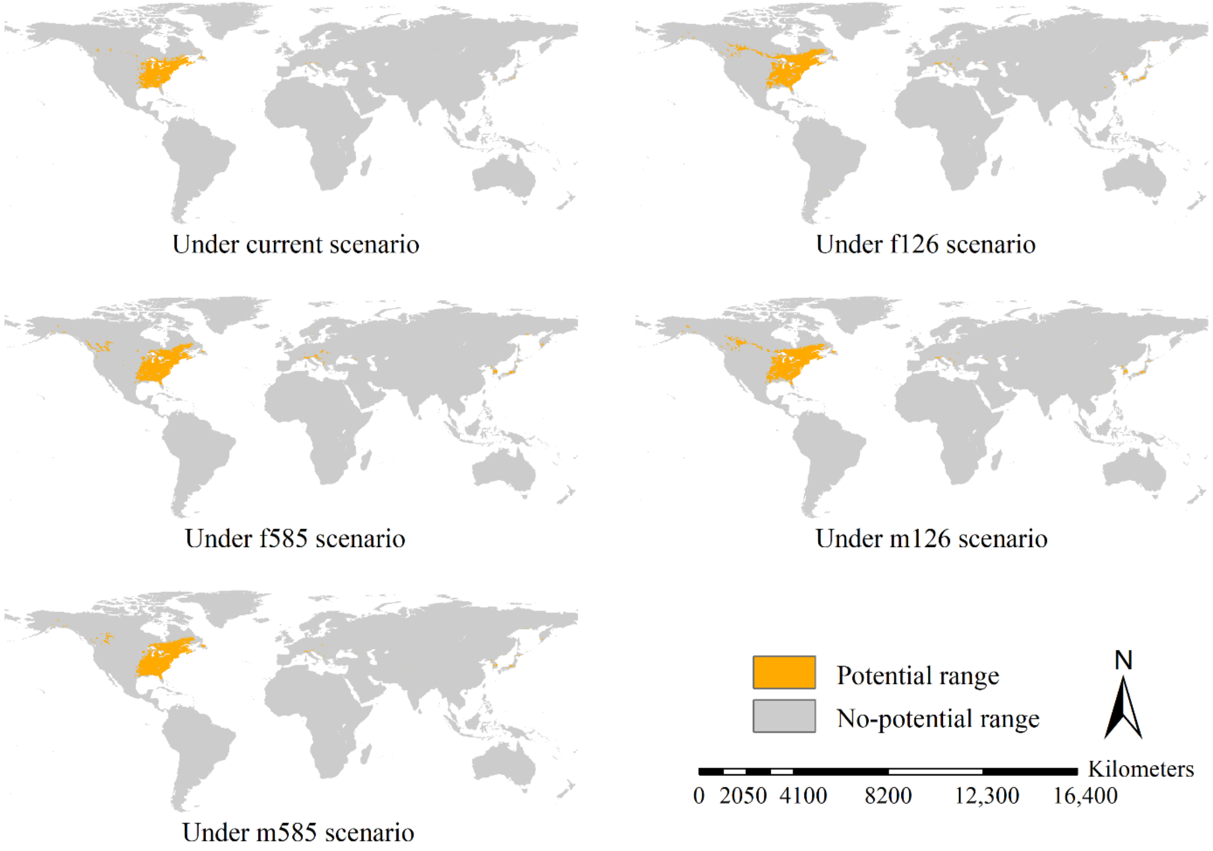

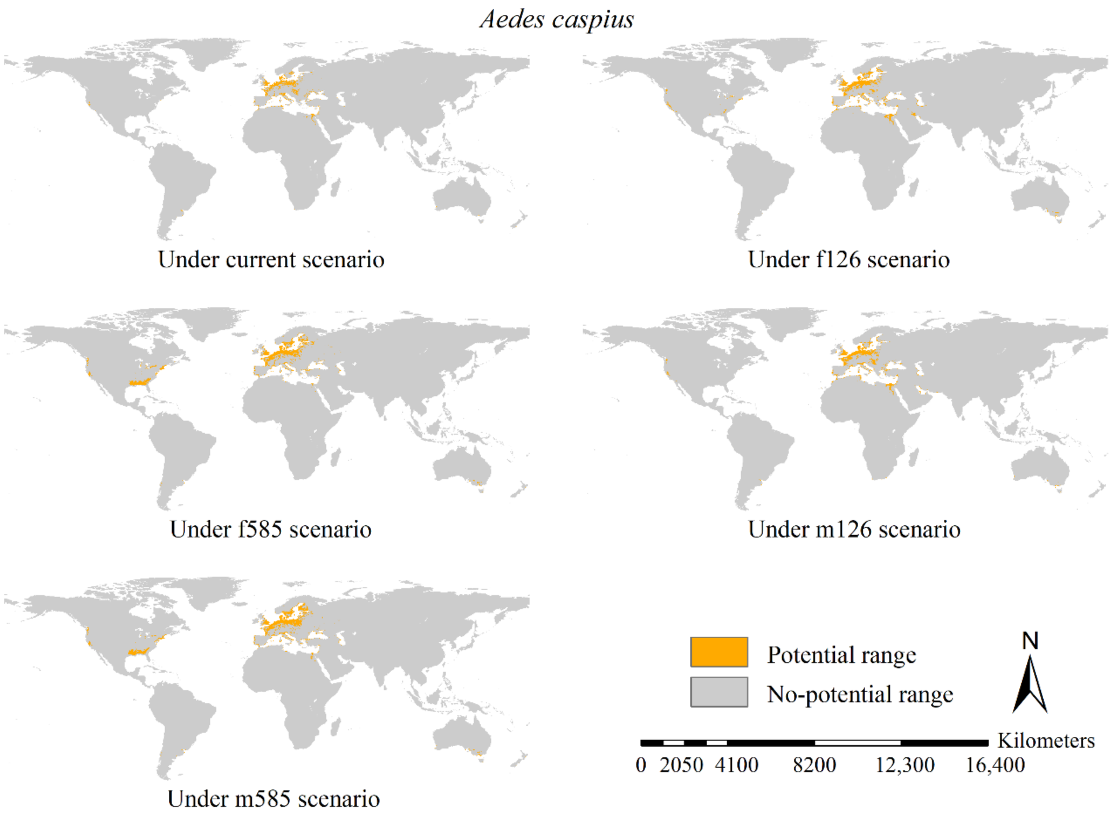
Aedes caspius
Under current scenario
Under f126 scenario
Under f585 scenario
Under m126 scenario
Under m585 scenario
Potential range
No-potential range
N
Kilometers
0 2050 4100 8200 12,300 16,400

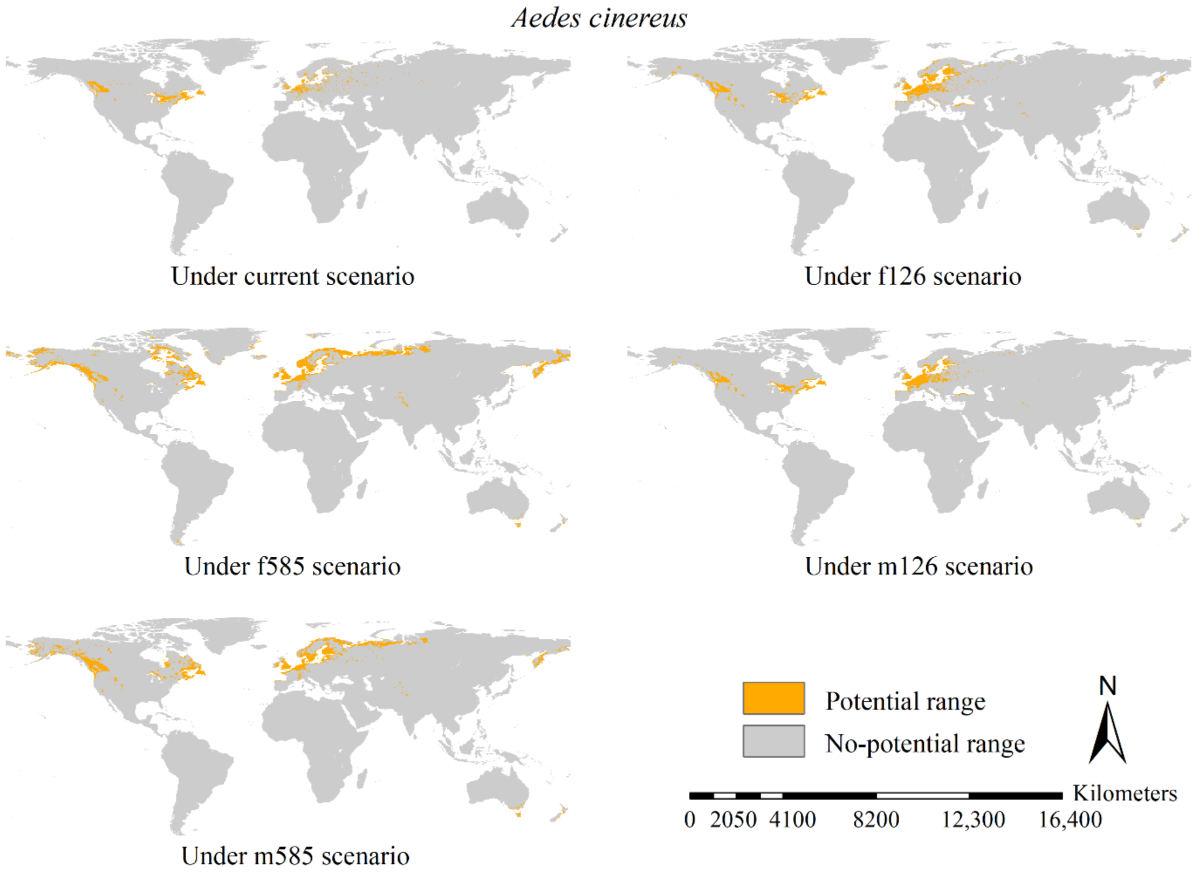
Aedes cinereus
Under current scenario
Under f126 scenario
Under f585 scenario
Under m126 scenario
Under m585 scenario
Potential range
No-potential range
N
Kilometers
0 2050 4100 8200 12,300 16,400

Aedes communis

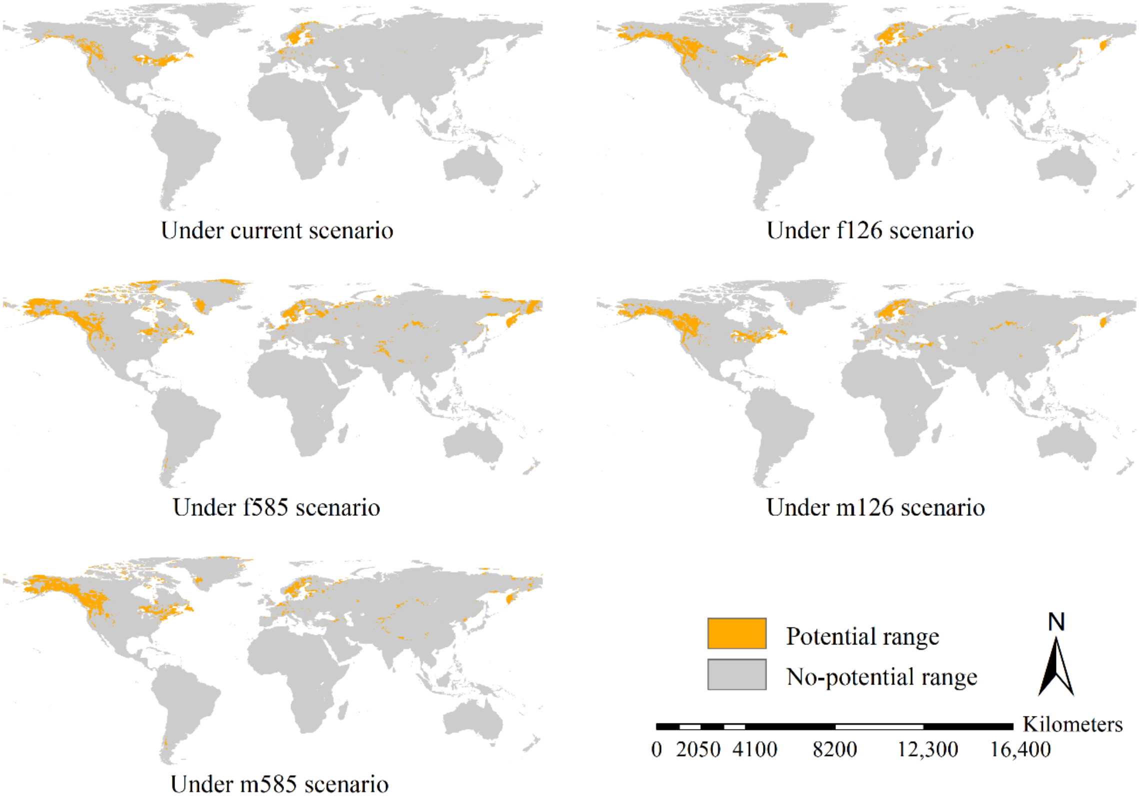

Aedes dorsalis

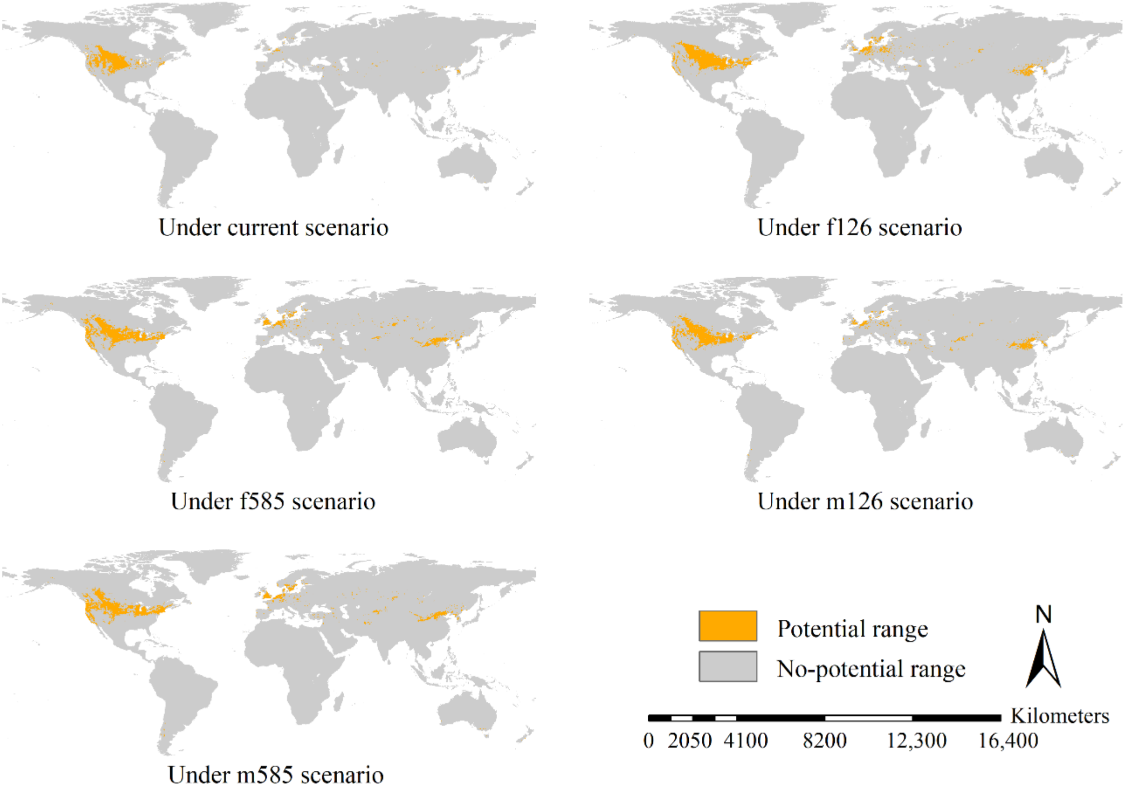

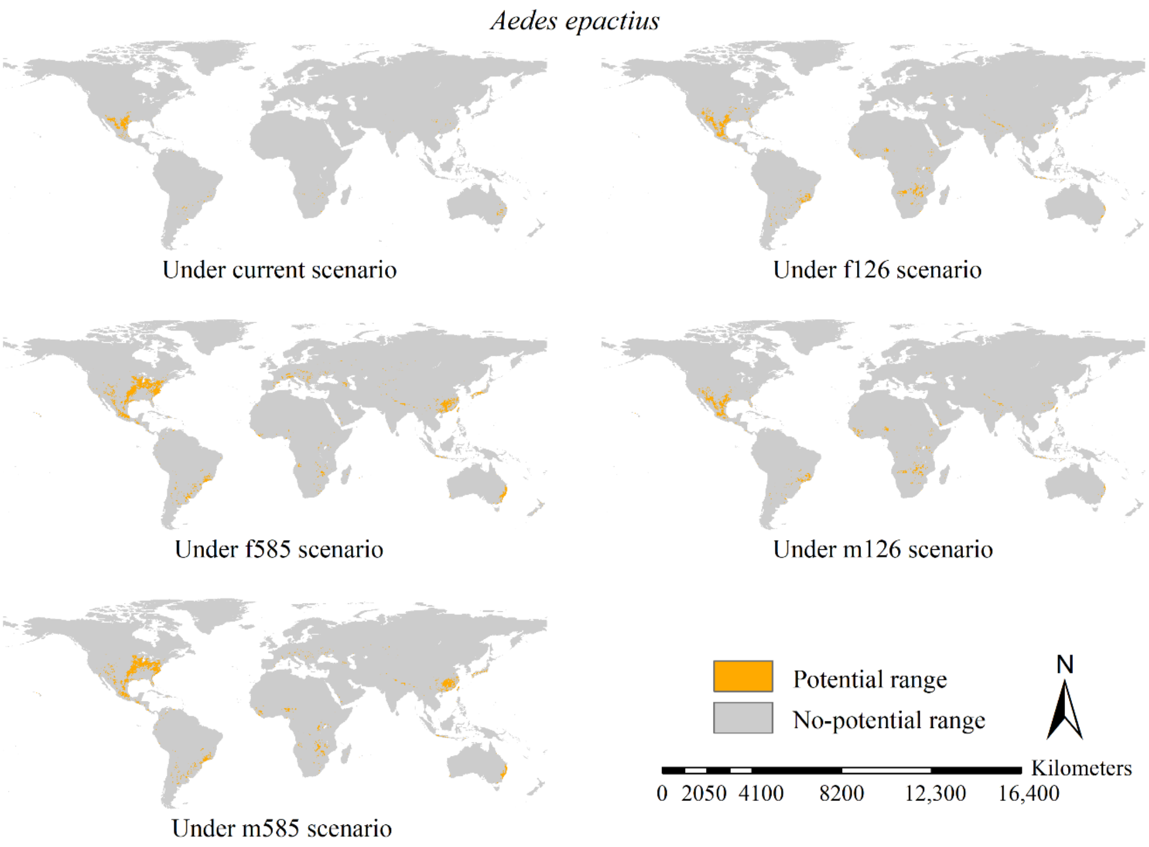
Aedes epactius
Under current scenario
Under f126 scenario
Under f585 scenario
Under m126 scenario
Under m585 scenario
Potential range
No-potential range
N
Kilometers
0 2050 4100 8200 12,300 16,400

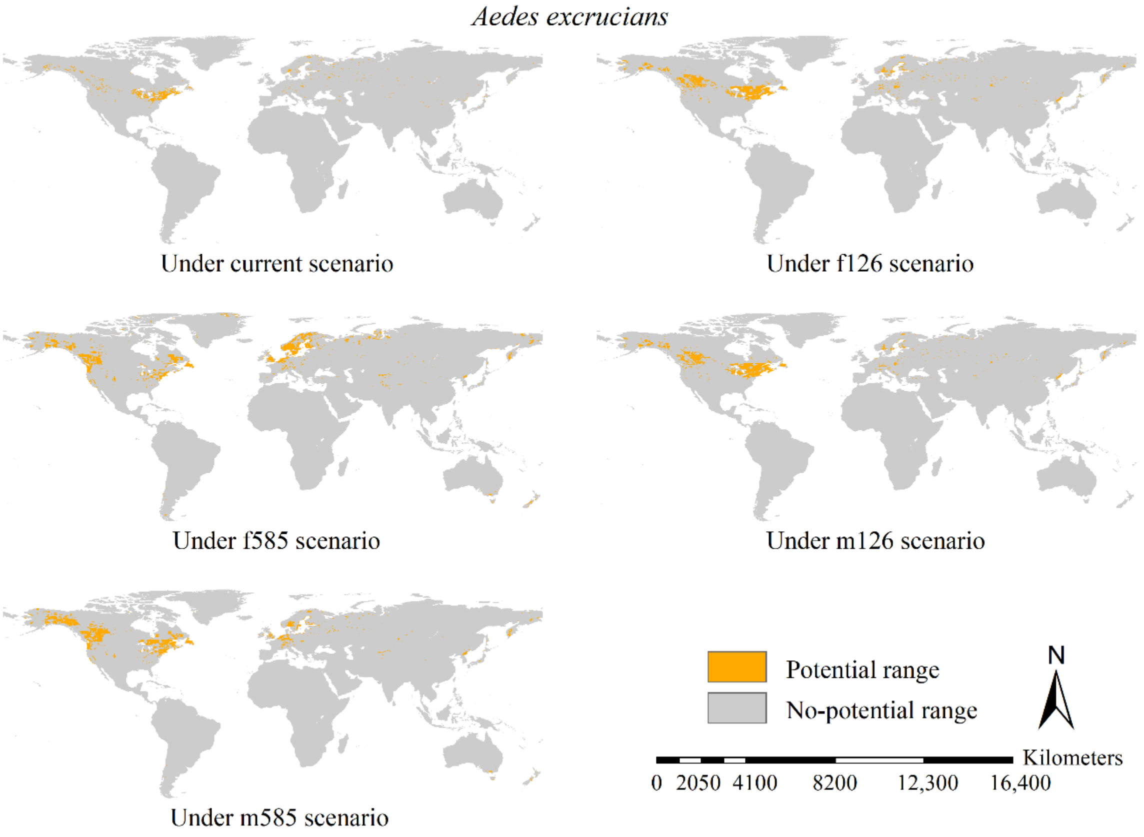
Aedes excrucians
Under current scenario
Under f126 scenario
Under f585 scenario
Under m126 scenario
Under m585 scenario
Potential range
No-potential range
N
Kilometers
0 2050 4100 8200 12,300 16,400

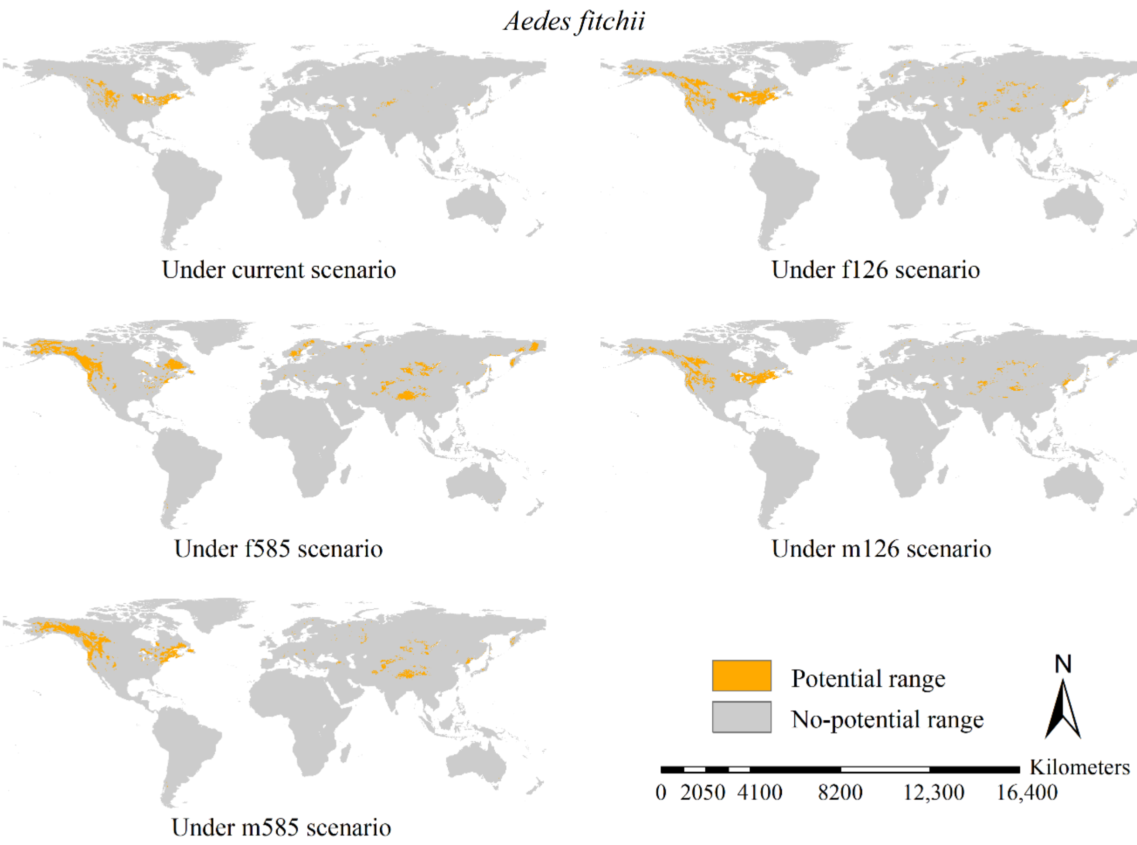

Aedes fitchii
Under current scenario
Under f126 scenario
Under f585 scenario
Under m126 scenario
Under m585 scenario
Potential range
No-potential range
N
Kilometers
0 2050 4100 8200 12,300 16,400

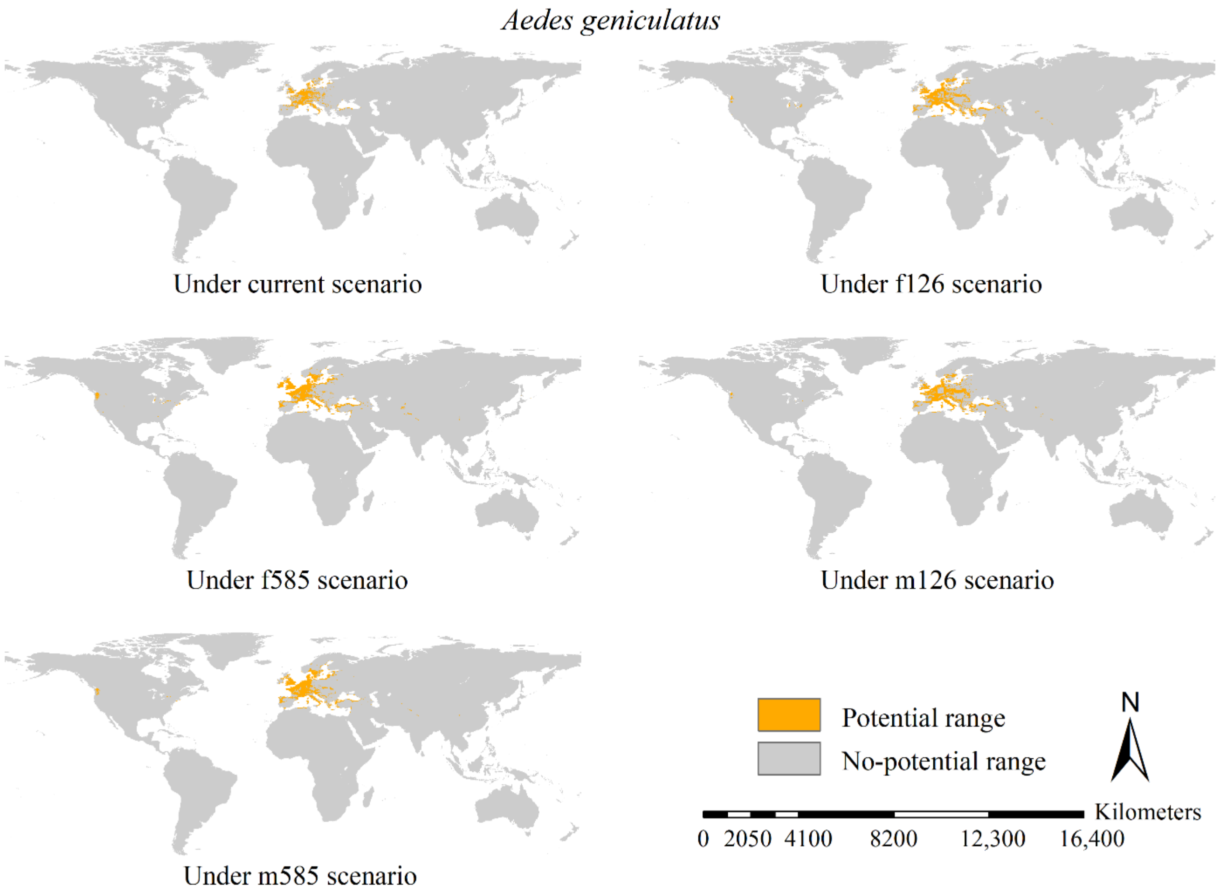

Aedes geniculatus
Under current scenario
Under f126 scenario
Under f585 scenario
Under m126 scenario
Under m585 scenario
Potential range
No-potential range
N
Kilometers
0 2050 4100 8200 12,300 16,400

Aedes intrudens

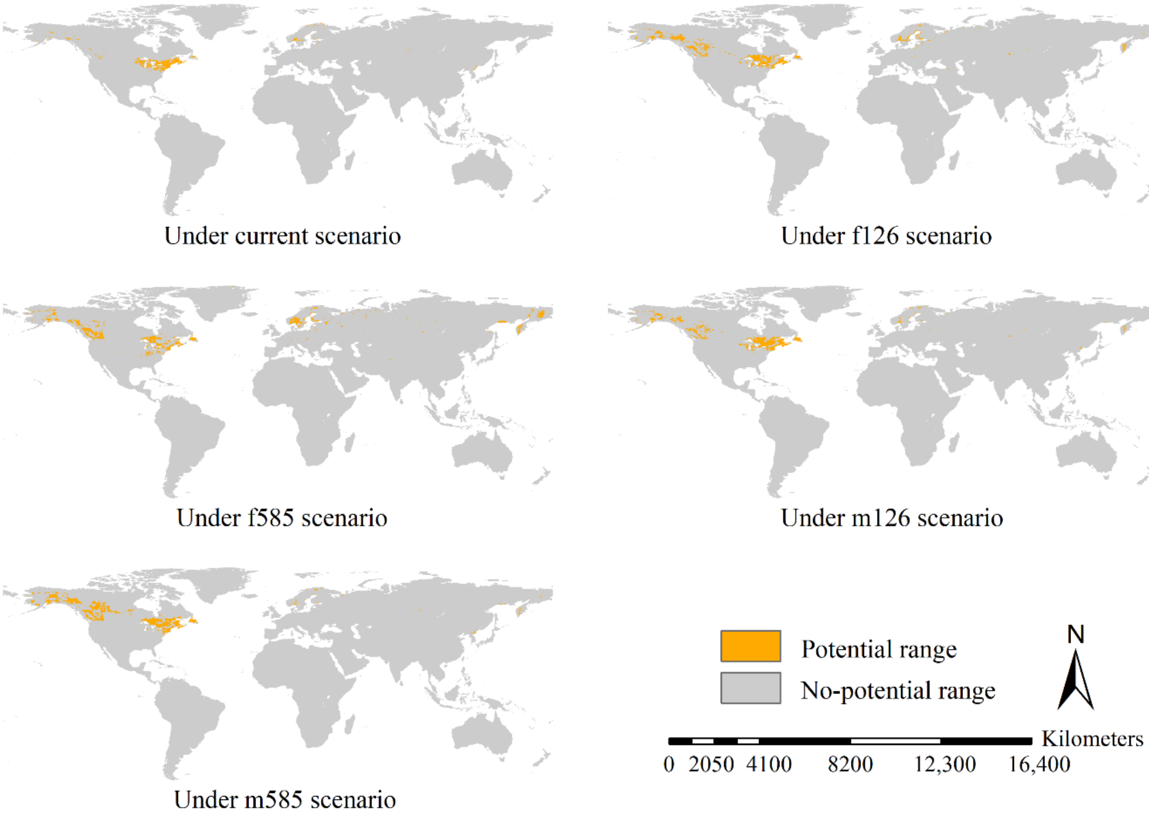

Aedes infirmatus

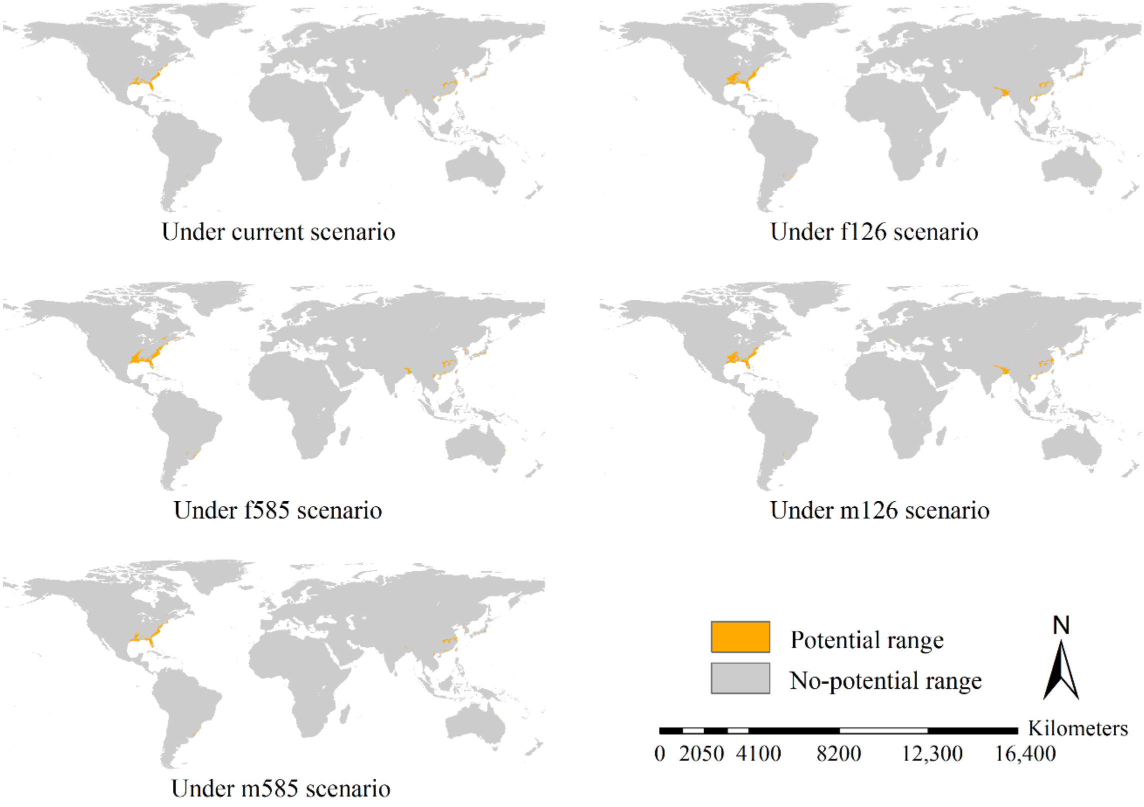

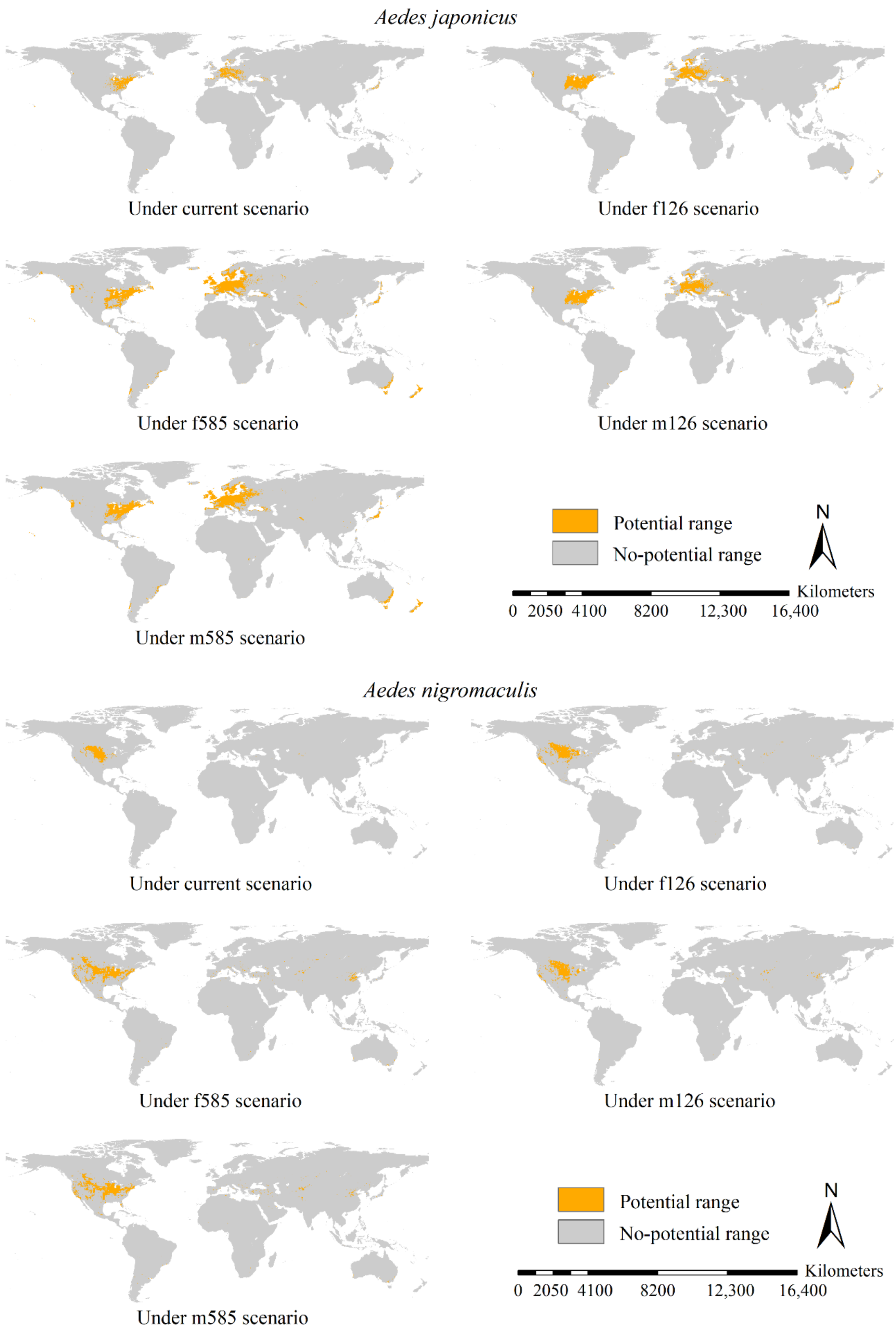
Aedes japonicus
Under current scenario
Under f126 scenario
Under f585 scenario
Under m126 scenario
Under m585 scenario
Potential range
No-potential range
N
Kilometers
0 2050 4100 8200 12,300 16,400
Aedes nigromaculis
Under current scenario
Under f126 scenario
Under f585 scenario
Under m126 scenario
Under m585 scenario
Potential range
No-potential range
N
Kilometers
0 2050 4100 8200 12,300 16,400

Aedes notoscriptus

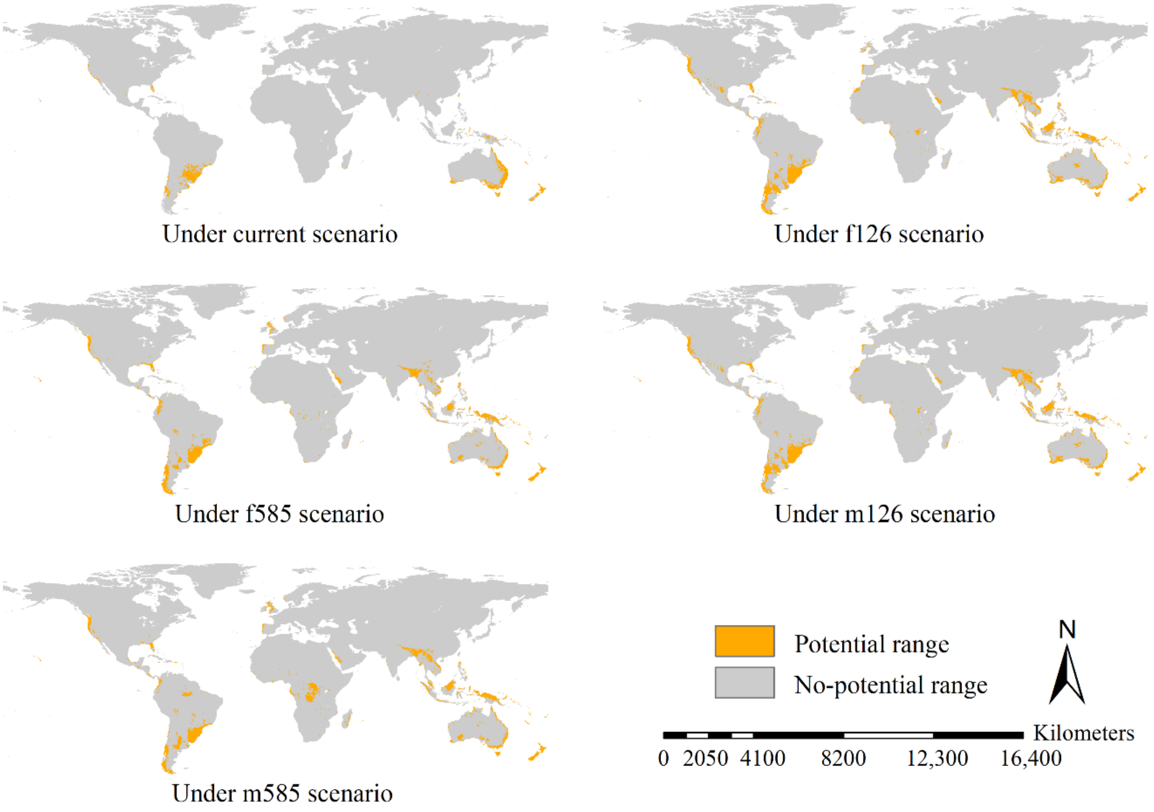

Aedes provocans

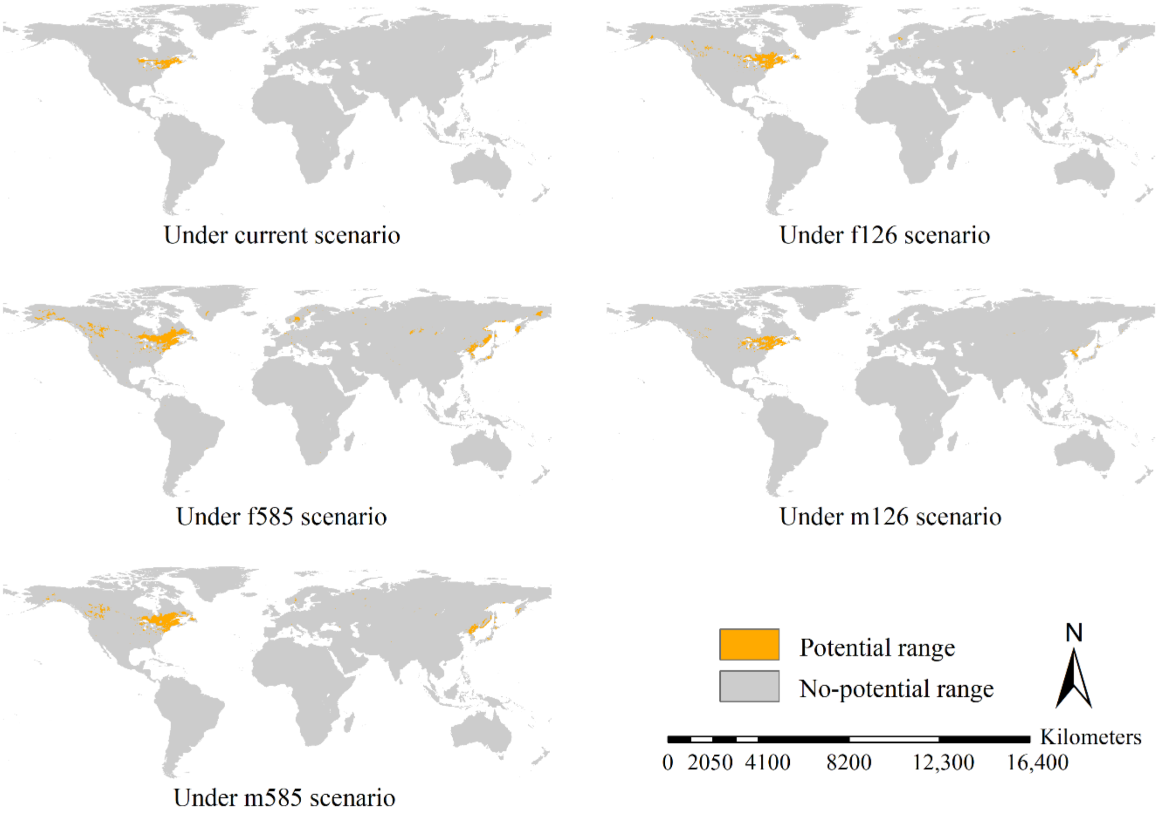

Aedes punctor

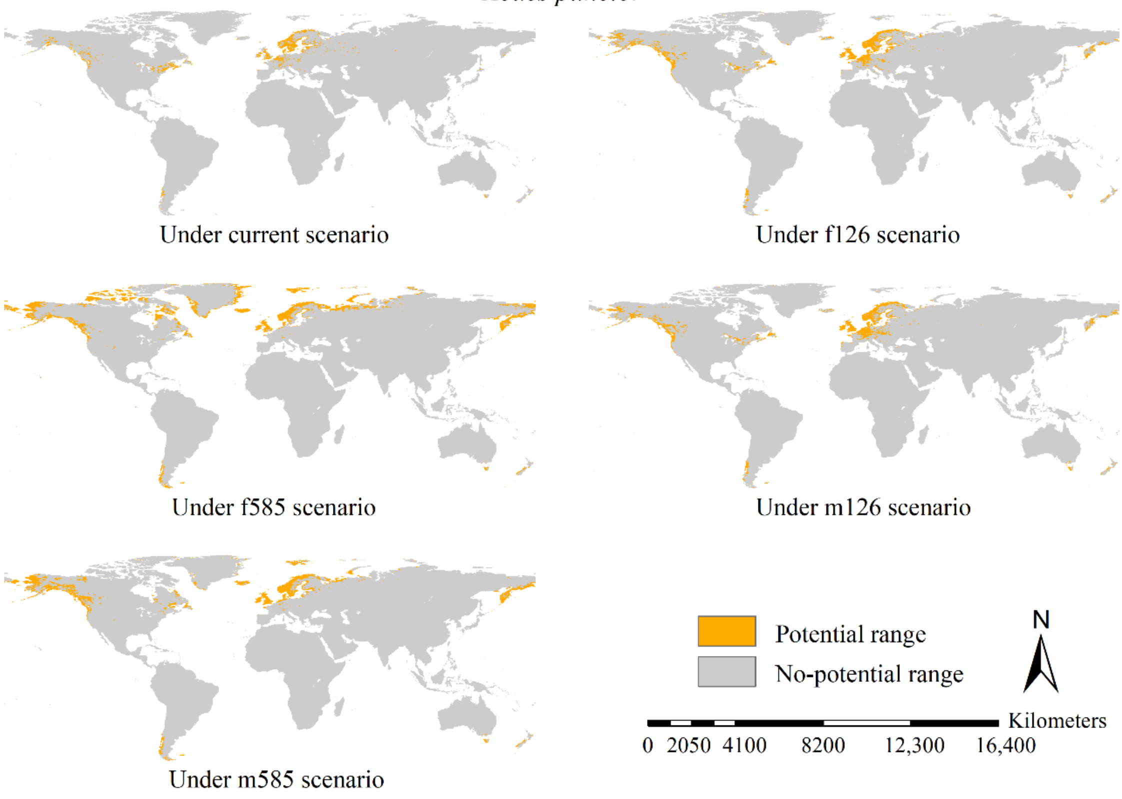

Aedes rubrithorax

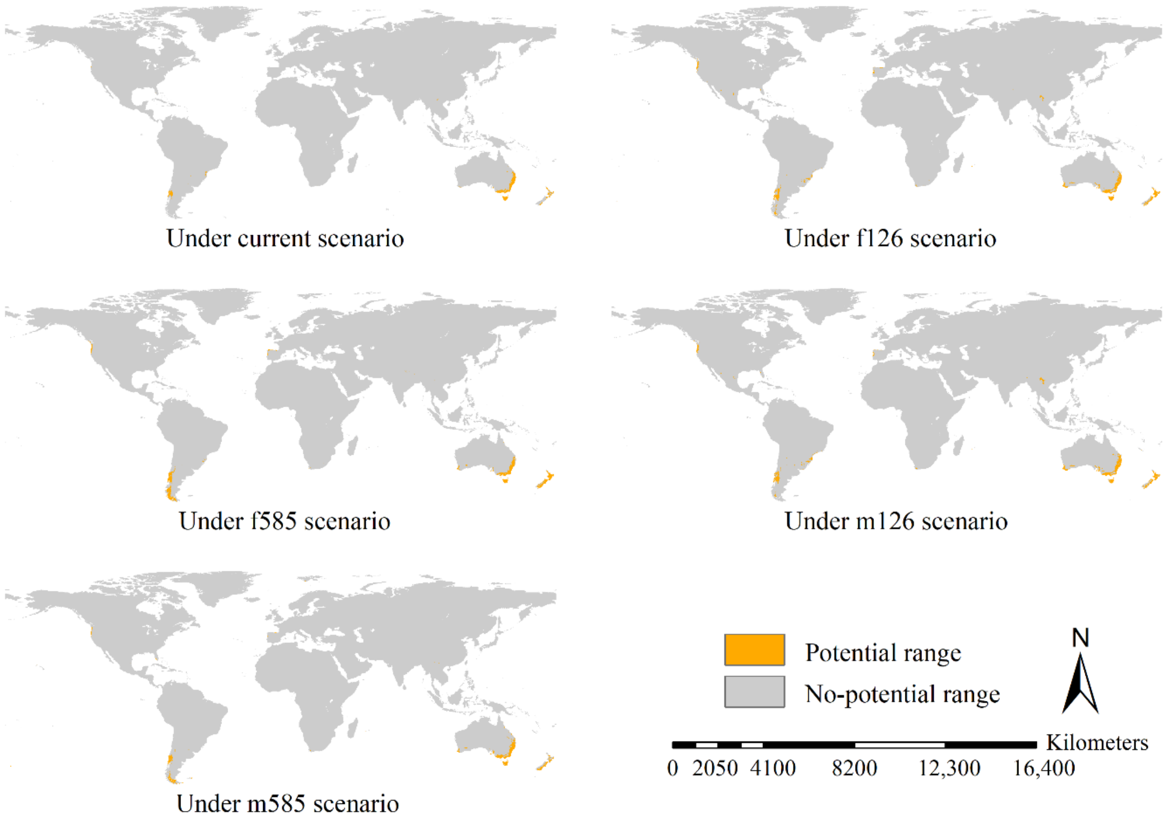

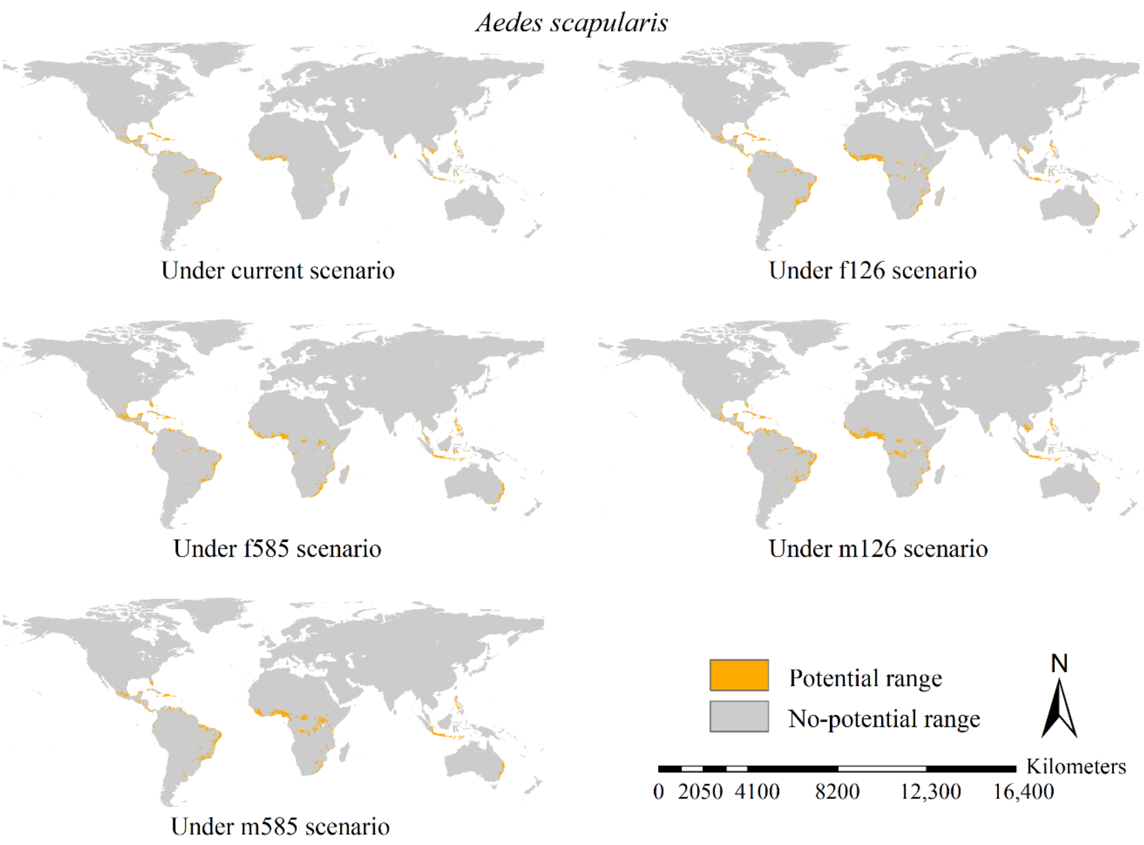
Aedes scapularis
Under current scenario
Under f126 scenario
Under f585 scenario
Under m126 scenario
Under m585 scenario
Potential range
No-potential range
N
Kilometers
0 2050 4100 8200 12,300 16,400

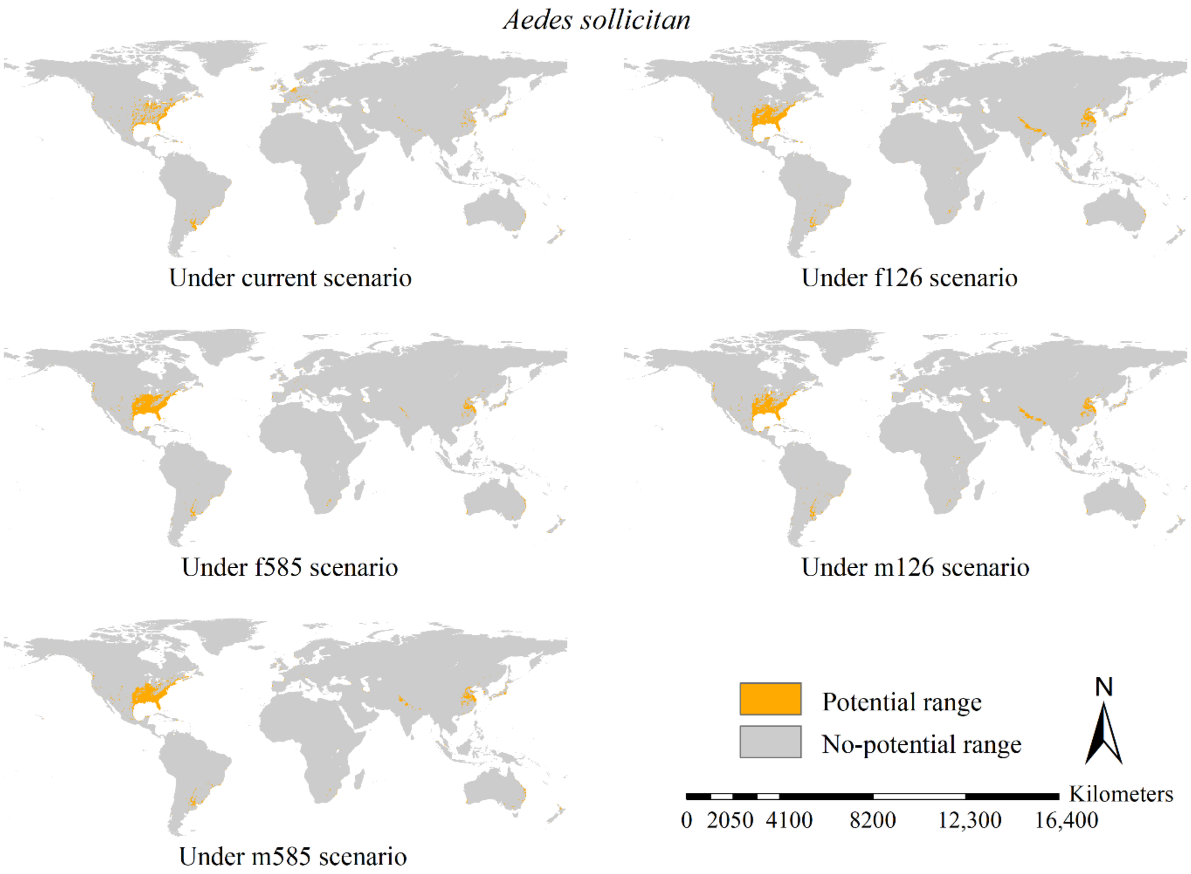
Aedes sollicitan
Under current scenario
Under f126 scenario
Under f585 scenario
Under m126 scenario
Under m585 scenario
Potential range
No-potential range
N
Kilometers
0 2050 4100 8200 12,300 16,400

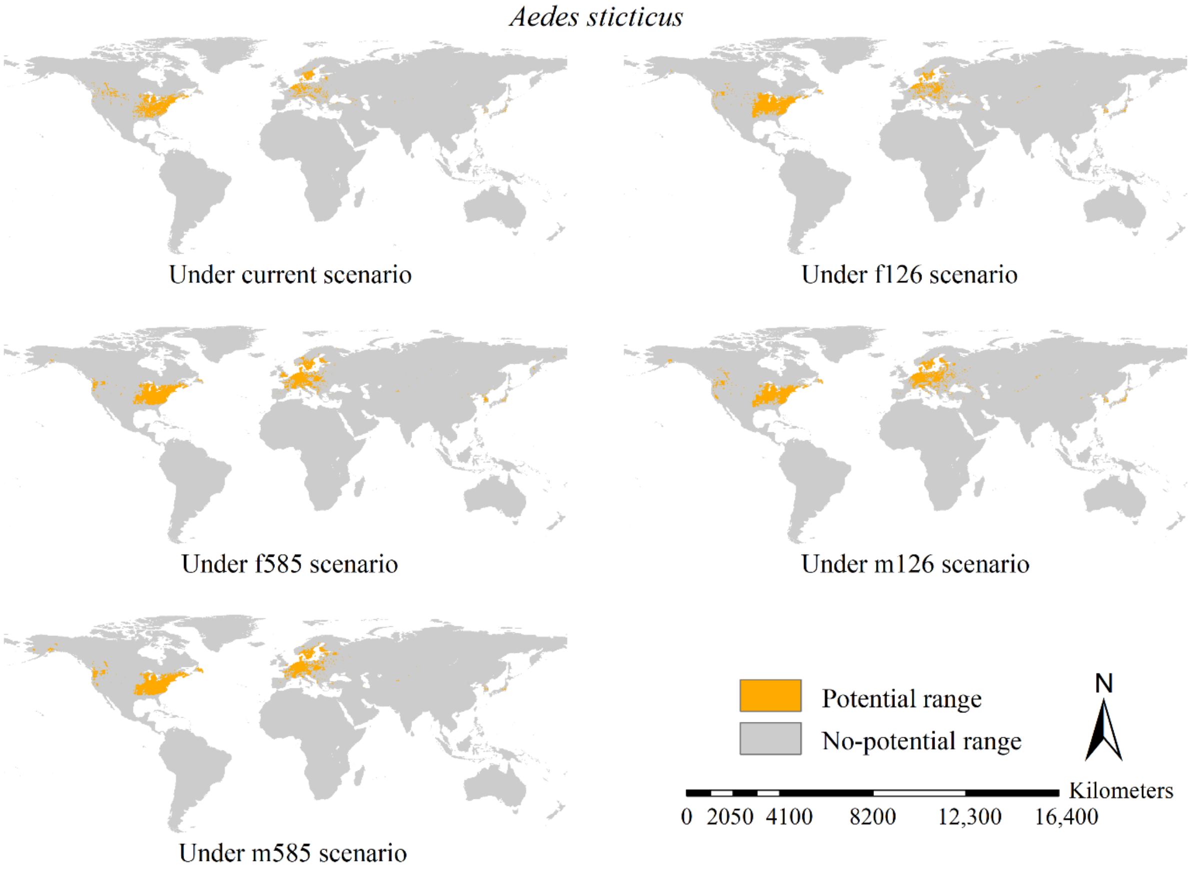
Aedes sticticus
Under current scenario
Under f126 scenario
Under f585 scenario
Under m126 scenario
Under m585 scenario
Potential range
No-potential range
N
Kilometers
0 2050 4100 8200 12,300 16,400

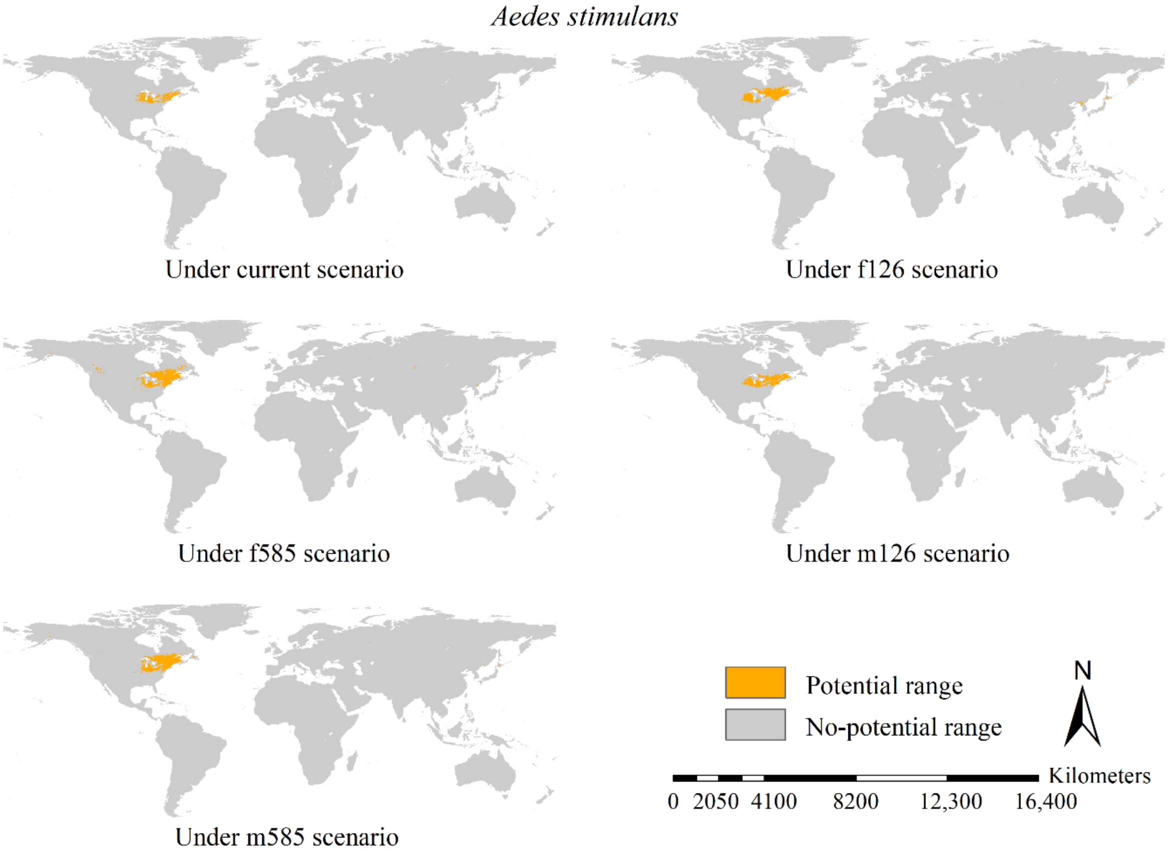
Aedes stimulans
Under current scenario
Under f126 scenario
Under f585 scenario
Under m126 scenario
Under m585 scenario
Potential range
No-potential range
N
Kilometers
0 2050 4100 8200 12,300 16,400

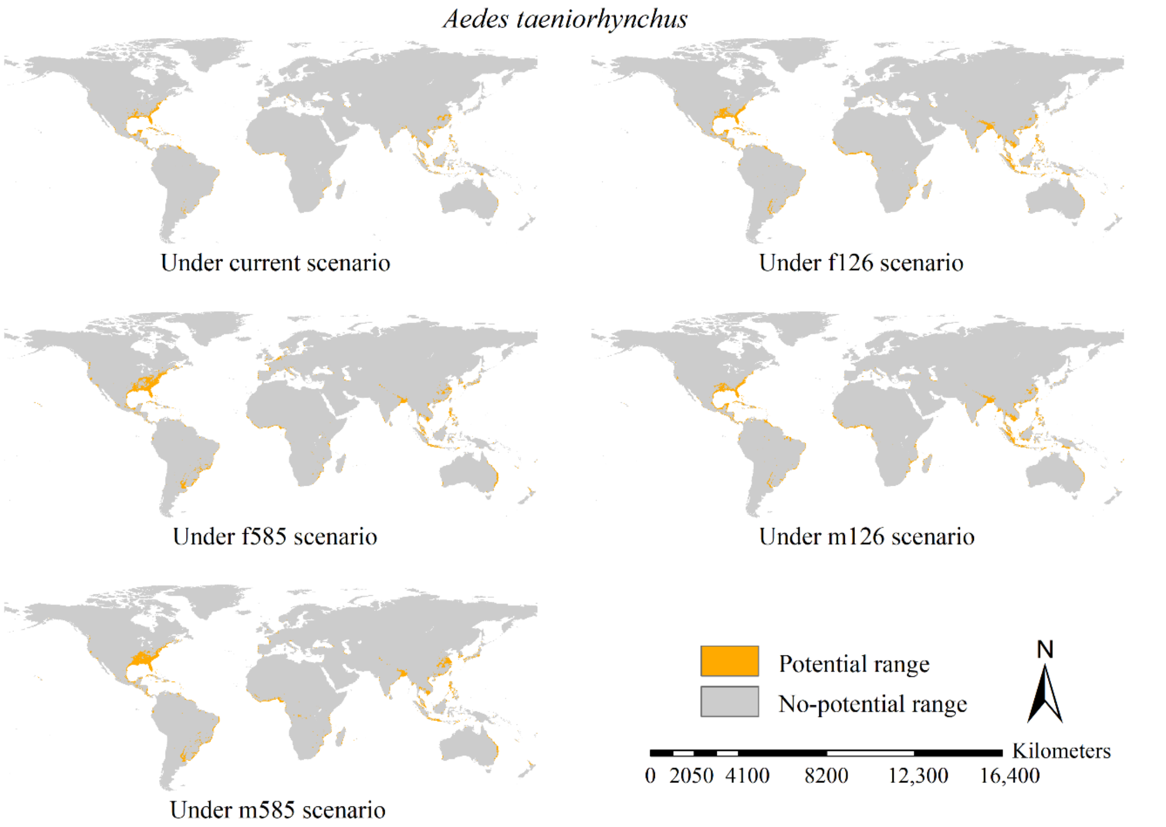
Aedes taeniorhynchus
Under current scenario
Under f126 scenario
Under f585 scenario
Under m126 scenario
Under m585 scenario
Potential range
No-potential range
N
Kilometers
0 2050 4100 8200 12,300 16,400

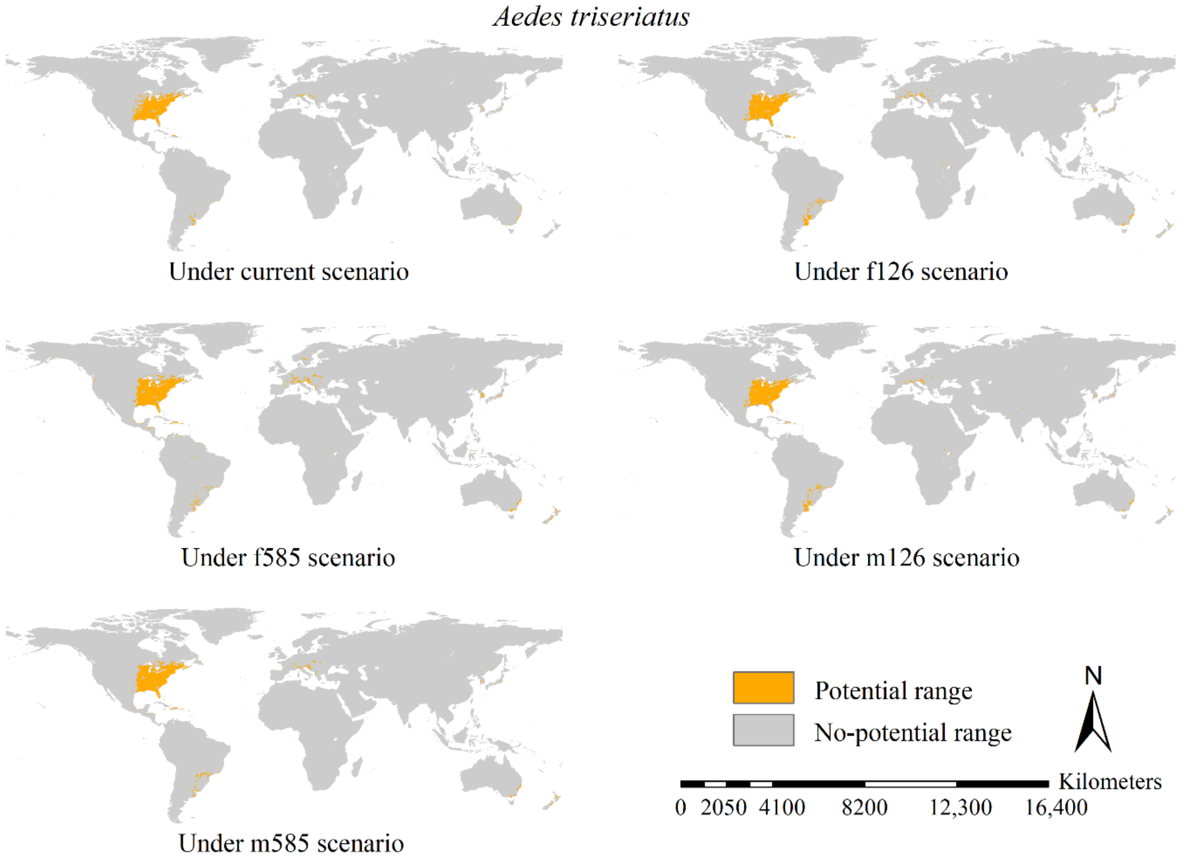
Aedes triseriatus
Under current scenario
Under f126 scenario
Under f585 scenario
Under m126 scenario
Under m585 scenario
Potential range
No-potential range
N
Kilometers
0 2050 4100 8200 12,300 16,400

Aedes trivittatus

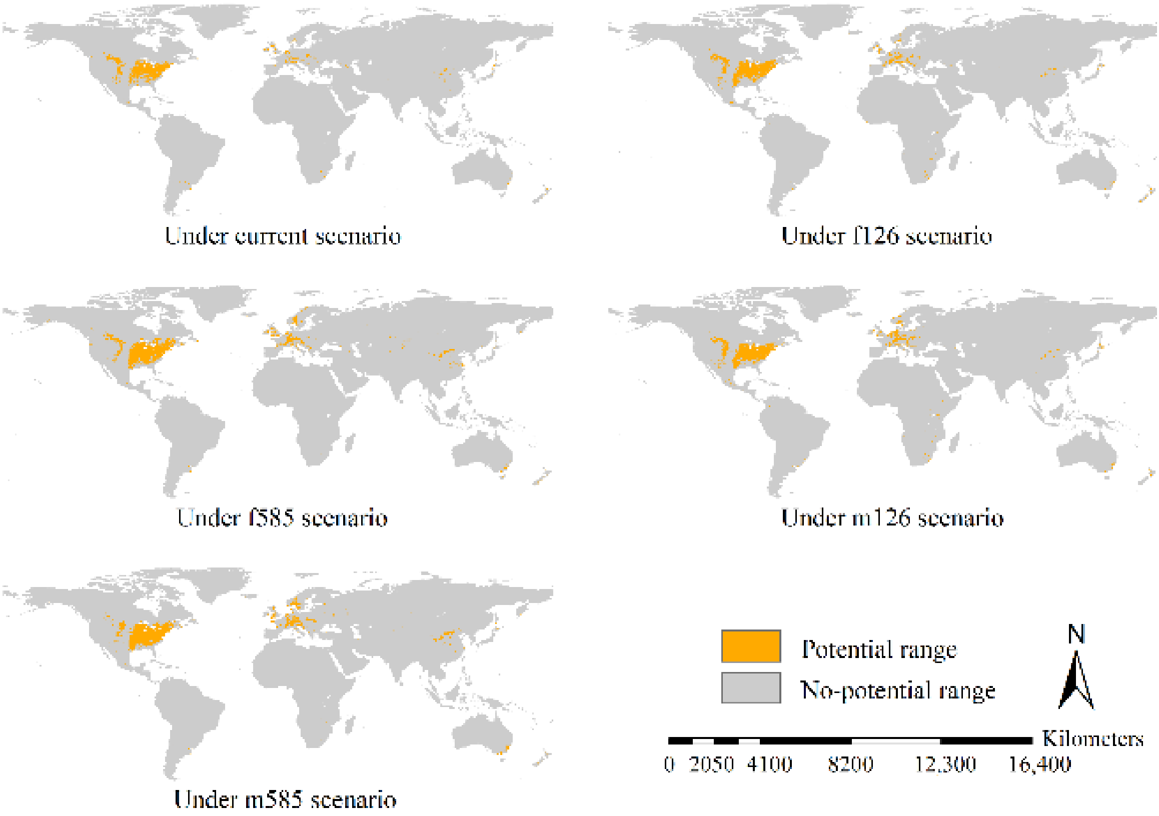

Aedes vexans

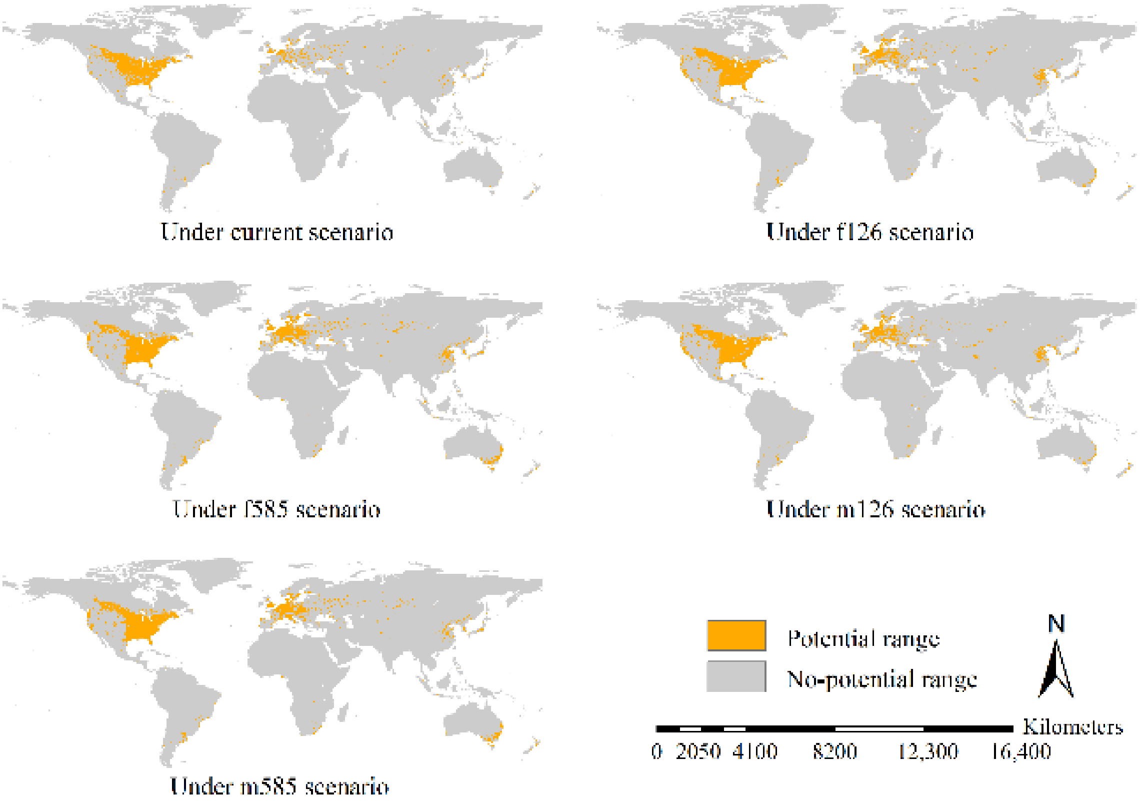

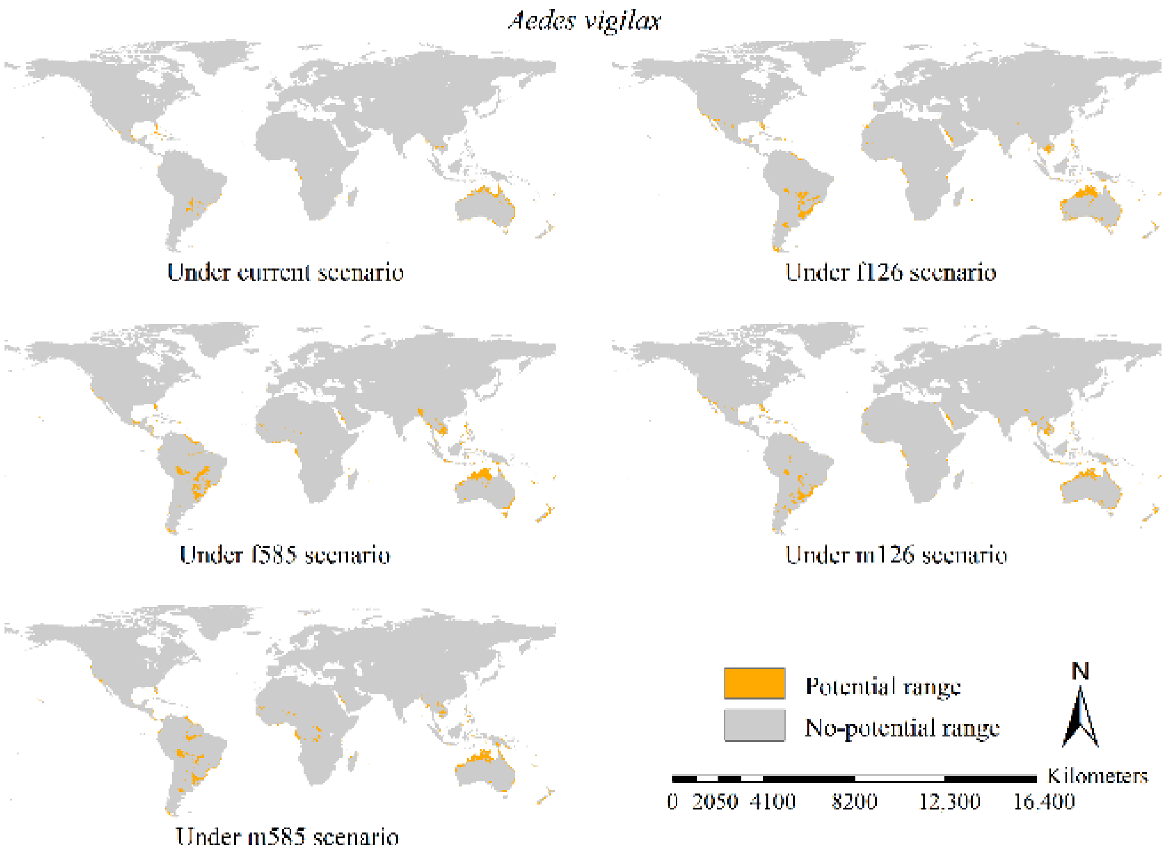
Aedes vigilax
Under current scenario
Under f126 scenario
Under f585 scenario
Under m126 scenario
Under m585 scenario
Potential range
No-potential range
N
Kilometers
0 2050 4100 8200 12,300 16,400
