# Supplementary material for: Future Climate Predicts Range Shifts and Increased Global Habitat Suitability for 29 Aedes Mosquito Species
Source: Insects. 2025 Apr 30;16(5):476. doi: 10.3390/insects16050476 (PMC12111898; doi:10.3390/insects16050476)
Supplement: Supplementary file 1 [file insects-16-00476-s001.zip › Supporting files/Table S6 MSS thresholds for determining the potential ranges.docx]

| Table S6 MSS thresholds for determining the potential ranges | | | | | |
| --- | --- | --- | --- | --- | --- |
|  | MSS threshold | | | | |
| Scientific name | Current | F126 | F585 | M126 | M585 |
| *Aedes aegypti* | 0.74 | 0.81 | 0.7 | 0.81 | 0.76 |
| *Aedes albopictus* | 0.70 | 0.78 | 0.73 | 0.79 | 0.75 |
| *Aedes atlanticus* | 0.84 | 0.83 | 0.80 | 0.84 | 0.81 |
| *Aedes canadensis* | 0.73 | 0.59 | 0.55 | 0.58 | 0.56 |
| *Aedes caspius* | 0.75 | 0.80 | 0.73 | 0.81 | 0.74 |
| *Aedes cinereus* | 0.72 | 0.65 | 0.45 | 0.70 | 0.52 |
| *Aedes communis* | 0.73 | 0.57 | 0.28 | 0.57 | 0.36 |
| *Aedes dorsalis* | 0.73 | 0.68 | 0.45 | 0.59 | 0.44 |
| *Aedes epactius* | 0.66 | 0.49 | 0.36 | 0.50 | 0.37 |
| *Aedes excrucians* | 0.67 | 0.59 | 0.29 | 0.61 | 0.38 |
| *Aedes fitchii* | 0.76 | 0.52 | 0.30 | 0.53 | 0.36 |
| *Aedes geniculatus* | 0.81 | 0.67 | 0.62 | 0.70 | 0.67 |
| *Aedes intrudens* | 0.69 | 0.65 | 0.43 | 0.68 | 0.54 |
| *Aedes infirmatus* | 0.71 | 0.71 | 0.75 | 0.70 | 0.71 |
| *Aedes japonicus* | 0.78 | 0.75 | 0.50 | 0.76 | 0.53 |
| *Aedes nigromaculis* | 0.78 | 0.58 | 0.40 | 0.62 | 0.38 |
| *Aedes notoscriptus* | 0.67 | 0.41 | 0.30 | 0.42 | 0.33 |
| *Aedes provocans* | 0.76 | 0.41 | 0.24 | 0.48 | 0.30 |
| *Aedes punctor* | 0.72 | 0.59 | 0.41 | 0.61 | 0.49 |
| *Aedes rubrithorax* | 0.72 | 0.55 | 0.48 | 0.55 | 0.54 |
| *Aedes scapularis* | 0.76 | 0.80 | 0.76 | 0.78 | 0.74 |
| *Aedes sollicitan* | 0.69 | 0.74 | 0.76 | 0.74 | 0.73 |
| *Aedes sticticus* | 0.73 | 0.73 | 0.60 | 0.68 | 0.61 |
| *Aedes stimulans* | 0.81 | 0.57 | 0.60 | 0.74 | 0.59 |
| *Aedes taeniorhynchus* | 0.72 | 0.67 | 0.62 | 0.69 | 0.63 |
| *Aedes triseriatus* | 0.74 | 0.76 | 0.73 | 0.77 | 0.75 |
| *Aedes trivittatus* | 0.70 | 0.75 | 0.70 | 0.73 | 0.70 |
| *Aedes vexans* | 0.72 | 0.78 | 0.72 | 0.77 | 0.73 |
| *Aedes vigilax* | 0.68 | 0.43 | 0.43 | 0.50 | 0.47 |
